# A marker weighting approach for enhancing within-family accuracy in genomic prediction

**DOI:** 10.1093/g3journal/jkad278

**Published:** 2023-12-11

**Authors:** Osval A Montesinos-López, Leonardo Crespo-Herrera, Alencar Xavier, Manje Godwa, Yoseph Beyene, Carolina Saint Pierre, Roberto de la Rosa-Santamaria, Josafhat Salinas-Ruiz, Guillermo Gerard, Paolo Vitale, Susanne Dreisigacker, Morten Lillemo, Fernando Grignola, Martin Sarinelli, Ezequiel Pozzo, Marco Quiroga, Abelardo Montesinos-López, José Crossa

**Affiliations:** Facultad de Telemática, Universidad de Colima, Colima, Colima, 28040, Mexico; International Maize and Wheat Improvement Center (CIMMYT), Km 45, Carretera México-Veracruz, CP 52640, Edo. de México, Mexico; Corteva Agrisciences, 8305 NW 62nd Ave, Johnston, IA 50131, USA; Purdue University, 915W State Street, West Lafayette, IN 47907, USA; International Maize and Wheat Improvement Center (CIMMYT), Km 45, Carretera México-Veracruz, CP 52640, Edo. de México, Mexico; International Maize and Wheat Improvement Center (CIMMYT), Km 45, Carretera México-Veracruz, CP 52640, Edo. de México, Mexico; International Maize and Wheat Improvement Center (CIMMYT), Km 45, Carretera México-Veracruz, CP 52640, Edo. de México, Mexico; Colegio de Postgraduados Campus, Tabasco, CP 86570, Mexico; Colegio de Postgraduados Campus Córdoba, Carretera Federal Córdoba-Veracruz km 348, Manuel León, Amatlán de los Reyes, Veracruz, CP 94953, Mexico; International Maize and Wheat Improvement Center (CIMMYT), Km 45, Carretera México-Veracruz, CP 52640, Edo. de México, Mexico; International Maize and Wheat Improvement Center (CIMMYT), Km 45, Carretera México-Veracruz, CP 52640, Edo. de México, Mexico; International Maize and Wheat Improvement Center (CIMMYT), Km 45, Carretera México-Veracruz, CP 52640, Edo. de México, Mexico; Department of Plant Science, Norwegian University of Life Sciences (NMBU), P.O. Box 5003, 1433 As, Norway; GDM Seed, Gibson City, IL, 60936, USA; GDM Seed, Gibson City, IL, 60936, USA; GDM, Chacabuco, Buenos Aires, B6740WAC, Argentina; GDM, San Isidro, Buenos Aires, B1642GLA, Argentina; Centro Universitario de Ciencias Exactas e Ingenierías (CUCEI), Universidad de Guadalajara, 44430, Guadalajara, Jalisco, Mexico; International Maize and Wheat Improvement Center (CIMMYT), Km 45, Carretera México-Veracruz, CP 52640, Edo. de México, Mexico; Colegio de Postgraduados, Montecillos, Edo. de México CP 56230, Mexico

**Keywords:** genomic prediction, adversarial validation, feature selection, leave one family out, within family genomic prediction

## Abstract

Genomic selection is revolutionizing plant breeding. However, its practical implementation is still very challenging, since predicted values do not necessarily have high correspondence to the observed phenotypic values. When the goal is to predict within-family, it is not always possible to obtain reasonable accuracies, which is of paramount importance to improve the selection process. For this reason, in this research, we propose the Adversaria-Boruta (AB) method, which combines the virtues of the adversarial validation (AV) method and the Boruta feature selection method. The AB method operates primarily by minimizing the disparity between training and testing distributions. This is accomplished by reducing the weight assigned to markers that display the most significant differences between the training and testing sets. Therefore, the AB method built a weighted genomic relationship matrix that is implemented with the genomic best linear unbiased predictor (GBLUP) model. The proposed AB method is compared using 12 real data sets with the GBLUP model that uses a nonweighted genomic relationship matrix. Our results show that the proposed AB method outperforms the GBLUP by 8.6, 19.7, and 9.8% in terms of Pearson’s correlation, mean square error, and normalized root mean square error, respectively. Our results support that the proposed AB method is a useful tool to improve the prediction accuracy of a complete family, however, we encourage other investigators to evaluate the AB method to increase the empirical evidence of its potential.

## Teaser

Within-family genomic prediction is fundamental in plant breeding. Montesinos-López *et al*. propose a method that combines the Adversarial Validation and the Boruta feature selection methods. This AB method builds a weighted genomic relationship matrix that is implemented with the Genomic Best Linear Unbiased Predictor (GBLUP) model. The authors compare the proposed AB method with the GBLUP model (C method) (no weighted genomic relationship matrix) and find that ABAB outperforms GBLUP in terms of genomic prediction accuracy.

## Introduction

Addressing the demands of a rapidly growing global population requires a focused effort on bolstering food production. However, achieving a substantial production increase is a complex endeavor, given a host of challenges. These encompass the depletion of natural resources, the scarcity of arable land, and the unpredictable shifts in climate patterns. Consequently, innovative strategies, such as the genomic selection (GS) methodology introduced by Meuwissen *et al*. (2001), have become indispensable for driving genetic advancements in vital crops such as wheat, rice, and maize. The application of GS holds the potential to fortify yield stability, increase productivity, bolster disease resistance, and enhance the nutritional and quality attributes of these essential crops (Crespo-Herrera *et al*. 2021).

Genomic selection stands as a transformative paradigm within both plant and animal breeding, capitalizing on high-density markers that span the genome's entirety. Its central premise revolves around the idea that genetic markers can (1) be in linkage disequilibrium with quantitative trait locus (QTL) responsible for a specific trait (Meuwissen *et al*. 2001) and (2) capture relationship patterns (Habier *et al*. 2010, 2013). Genomic selection is redefining breeding practices through an array of innovative mechanisms, encompassing (1) proactive genotype identification, (2) heightened selection precision, (3) resource optimization, (4) accelerated variety development, (5) intensified selection efforts, (6) assessment of complex traits, and (7) enhanced selection accuracy. In sum, genomic-assisted breeding selection represents a paradigm shift in both plant and animal breeding, reshaping the landscape through predictive analysis and precision driven selection; its versatility and merits position GS as a potent instrument propelling progress and ingenuity in agricultural enhancement endeavors.

The challenge of achieving accurate genomic predictions stems from the intricate nature of genomic data and the intricate interplay between genes and traits. While GS holds the potential to revolutionize breeding strategies, the precise prediction of complex traits based solely on genetic markers presents a formidable complexity. The polygenic nature of numerous agricultural traits adds to the complexity, as multiple genes with subtle effects collectively contribute to the phenotype. Additionally, the accuracy of predictions is intricately tied to the quality and representativeness of the training population used to construct prediction models. Inadequate or biased data can lead to suboptimal predictions and hinder breeding progress. Genetic interactions and environmental influences further complicate genomic predictions, as these interactions often remain incompletely understood or challenging to incorporate into predictive models. Consequently, ongoing research, advancements in data analysis techniques, improved genotyping technologies, and a deeper understanding of genotype–phenotype relationships are pivotal to address these challenges and fully unlock the potential of genomic prediction within agricultural breeding programs.

In the search to enhance the prediction accuracy of the GS methodology, various modeling approaches have been proposed. These ranges from Bayesian frameworks encompassing the Bayesian alphabet (BayesA, BayesB, BayesC, and Bayesian Lasso, etc.) to machine learning methodologies such as random forest, support vector machine, gradient boosting machine, and deep neural networks for genomic prediction. Nonetheless, mixed models remain prevalent in genomic prediction due to their reliable performance in terms of prediction accuracy, computational efficiency, interpretability, and straightforward tuning processes.

However, despite the considerable promise held by GS and the diverse modeling approaches explored so far, translating these concepts into practical implementation remains a complex challenge. This complexity arises from the multitude of factors that must be optimized to achieve high prediction accuracies. The simultaneous optimization of these factors introduces intricacy, often yielding unforeseen outcomes. Consequently, the successful integration of GS into real-world scenarios calls for ongoing research, robust methodologies, and a comprehensive understanding of the intricate interactions underlying these contributing factors. Addressing these challenges is imperative to fully realize the potential of GS and facilitate its seamless integration into breeding programs and agricultural practices (Montesinos-López *et al*. 2022).

Precisely predicting family performance is a cornerstone of effective plant breeding, enabling the maximization of genetic gains, efficient allocation of resources, strategic parental selection, and the tailoring of breeding strategies. Furthermore, it enhances disease resistance, adaptability, end-use quality, and consistency in crop varieties. Accurate family prediction empowers breeders to channel their efforts toward superior families, leading to the development of enhanced and resilient crop varieties with desirable traits.

When crossing two diploid organisms, each offspring inherits one allele from each parent, and the selection of which specific allele an offspring inherits occurs through a stochastic process. This process creates genetic diversity within a family, a phenomenon referred to as Mendelian segregation variance or within-family variance. In statistical terms, the genetic value of a quantitative trait in an offspring is composed of the four alleles contributed by the two parents, guided by a Mendelian variable from each parent. Wang and Xu (2019) proposed a mixture model that allows for the dissection of the total genetic variance into between-family variance and within-family variance. With no inbreeding, the genetic variance is evenly split between-family variance and within-family variance. When inbreeding is present, there is an increase in the overall genetic variance due to an increase between-family variance at the expense of the within-family variance (Foulley and Chevalet 1981).

However, predicting family performance within plant breeding is fraught with complexities stemming from factors such as pronounced genetic diversity, intricate trait architectures, limited family sizes, erratic environmental variations, genotype-by-environment interactions (GE), data quality and quantity challenges, trait trade-offs, and extended breeding cycles. Overcoming these challenges requires advanced methodologies, refined technologies, expansive datasets, and a profound understanding of intricate trait genetics. Despite these challenges, accurate family prediction is pivotal for the development of superior crop varieties and to address global agricultural challenges.

The interplay of genetic diversity, recombination, genetic segregation, population-specific variation, incomplete data, environmental elements, and statistical intricacies collectively underscore the challenges associated with genomically predicting full siblings resulting from a single cross. While advancements in genomics and statistical methodologies continue to enhance prediction accuracy, achieving precise predictions of genetic relatedness remains a multifaceted endeavor, particularly when working with limited data from a single cross. Predicting full siblings from a single cross requires the handling of intricate relationships and uncertainties, further intensifying the complexity of the analysis and prediction processes.

As such, innovative methodologies to enhance family prediction accuracy are indispensable. In this pursuit, we explore the fusion of the adversarial validation (AV) approach proposed by Montesinos-López, Kismiantini *et al*. (2023) with the Boruta feature selection method, as examined by Montesinos-López, Crespo-Herrera *et al*. (2023). Although the AV method adeptly detects training-testing mismatches and optimizes training sets, its efficiency may diminish when dealing with smaller training sets or moderate to high mismatches. Conversely, the Boruta method, a feature selection technique, effectively mitigates prediction errors by selecting a fraction of the most important features, but may not consistently elevate selection accuracy, since when the selected variables are not optimal, decreases prediction accuracy.

Thus, in this research, we introduced the AB method, which leverages the strengths of both AV and Boruta. The AB method primarily functions to mitigate disparities between training and testing distributions without reducing the training set and original inputs. This is achieved through the attenuation of weights assigned to markers that manifest the most substantial differences between the training and testing sets. In the AB method, a binary classifier is trained for each family using a combination of original and shuffled markers. Feature importance scores for the original markers are computed, and these weighted markers are employed to construct a genomic relationship matrix (GRM). This weighted GRM is then incorporated into the genomic best linear prediction model (GBLUP), forming the core of the AB method.

We hypostatize that the AB method holds the potential to enhance prediction accuracy and mitigate prediction errors, particularly in cases where a substantial disparity exists between the training and testing sets. Conversely, in scenarios where such a discrepancy is inconsequential, the AB will not affect the prediction performance. This assertion is grounded in the contention that the AB method addresses a notable limitation of the AV method (Montesinos-López, Kismiantini *et al*. 2023). Specifically, the AV method struggles when confronted with small datasets, either failing to identify an optimal training set or, when the dataset is so diminutive, adversely impacting prediction performance. In contrast, the AB method using all original inputs with appropriate weighting, circumvents these issues. By eschewing the selection of a mere sample or fraction of inputs, as seen in variable selection methods (Boruta method), the AB method sidesteps the pitfalls associated with a suboptimal sample of inputs, which can detrimentally affect prediction performance. We assess the AB method against conventional GRMs without weights (GBLUP), utilizing 12 real-world datasets.

## Materials and methods

### GBLUP model

The model used in this study can be represented as


(1) $$Y_{ij}=\mu +E_{i}+g_{j}+gE_{ij}+\epsilon_{ij},$$

where $Y_{ij}$ denotes the response variable in the environment *i* and genotype *j*. $E_{i}$ are the fixed effects of environments, $g_{j},$j=1,…,J, denotes the random effects of lines, $gE_{ij}$ denotes the random effects of the genotype-by-environment interaction modeled through a compound symmetry structure, and $\epsilon_{ij}$ denotes the random error components in the model assumed to be independent normal random variables with mean 0 and variance $\sigma^{2}$. Furthermore, it is assumed that $g=(g_{1},\ldots ,g_{J}{)}^{T}∼N_{J}(0,\sigma_{g}^{2}G)$, $gE=(gE_{11},\ldots ,gE_{1J},\ldots ,\;gE_{IJ}{)}^{T}∼N_{IJ}(0,\sigma_{gL}^{2}(Z_{E}Z_{E}^{T}⊙Z_{g}{GZ}_{g}^{T}))$, where $Z_{E}$ is the design matrix of environments of order n×I, ⊙ denotes the Hadamard product, and $\;Z_{g}$ is the design matrix of genotypes (lines) of order n×J,G is the genomic relationship-matrix computed using markers (VanRaden 2008). Let X denote the matrix of markers and let M, be the matrix of centered and standardized markers. Then $G=\frac{MM^{T}}{p}$ (VanRaden 2008), where *p* is the number of markers. The implementation of this model (1) using the computed G was done in the Bayesian Genomic Linear Regression library of Pérez and de los Campos (2014).

### Feature selection—Boruta algorithm

The Boruta algorithm was developed to identify covariates that have significant relevance to the response variable, whether strong or weak. Specifically designed for high-dimensional datasets with noisy features, Boruta addresses the challenges of feature selection in such datasets (Kursa and Rudnicki 2010). Its operation involves the creation of a shadow (permuted) feature set, which is a replicated version of the original feature set with values randomly permuted, i.e. shuffled. These shadow features act as controls to assess the statistical significance of the original features. The determination of the relevance of the original features is based on whether their importance scores significantly surpass the importance scores of their corresponding shadow features.

In datasets containing numerous noisy features, where conventional feature selection methods may face difficulties, Boruta proves to be an efficient solution. However, it is important to note that it can be computationally intensive and requires careful parameter tuning to achieve optimal results (Kursa and Rudnicki 2010).

The Boruta algorithm operates through a series of well-defined steps for effective feature (marker in our application) selection. The process is as follows:

Step 1: A shadow feature set is generated by randomly permuting the values of each feature in the original dataset.

Step 2: A random forest model is trained using both the original feature set and the shadow feature set. This model serves as the foundation for assessing feature importance.

Step 3: The feature importance scores for each original feature are calculated by comparing them to the importance scores of their corresponding shadow features.

Step 4: The maximum importance score is determined for each feature based on the results obtained in Step 3.

Step 5: The Binomial test is employed to evaluate the statistical significance of each feature. If a feature's importance score is deemed statistically significant, it is marked as “important”; otherwise, it is labeled as “unimportant.” The Binomial test is a statistical evaluation used in Boruta to compare the observed number of successes (e.g. instances where a feature's importance score exceeds a certain threshold) with the expected number of successes under a null hypothesis. This test determines whether the observed results are statistically significant or merely due to chance. In Boruta, the Binomial test is applied to assess whether the feature importance scores are significantly higher than those of the shadow features, thereby indicating the relevance of the original features (Kursa and Rudnicki 2010).

Step 6: The process outlined in Steps 1–5 is repeated for a predetermined number of iterations to ensure robustness and consistency in the feature selection process.

Step 7: Finally, the features are ranked based on their importance scores. Within Boruta, features are categorized as “Confirmed” if they are considered important, “Rejected” if they are deemed unimportant, and “Tentative” if further investigation is required or if they are considered less important. This categorization provides valuable insights into the relevance and contribution of each feature to the predictive model.

## The proposed AB method

Step 1. We assume that we have a multi-environment or single environment data set in which there are at least 2 families and for each family there are some lines. Since our goal is to predict a complete family, the information of this family constitutes the testing set { $X_{tst}$, $y_{tst}$}, and the information of the remaining families is the training set { $X_{trn}$, $y_{trn}$}. Then, in the original dataset { $X_{trn}$, $y_{trn}$, $X_{tst}$, $y_{tst}$}, we remove the original response variable column { $y_{trn}$, $y_{tst}$}, and add a fictitious (new) response variable column { $y_{f\_trn}$, $y_{f\_tst}$} that replaces the source of the data by 0 (that is, $y_{f}=0$) for samples (observations) on the training set and by 1 (i.e. $y_{f}\;=1$) for the samples in the testing set (Montesinos-López, Kismiantini *et al*. 2023). In other words, the fictitious response variables with 0 s correspond to the remaining families and the 1 s for the family we want to predict.

Step 2. We then implement the Boruta algorithm with inputs { $X_{trn}$, $y_{f\_trn}$, $X_{tst}$, $y_{f\_tst}$} and we extract the variable important scores (IS) for each marker.

Step 3. The next step is to compute the inverse of the IS as Inv_IS = 1/IS, then compute the final weights as **w** = Inv_IS ×p /sum(Inv_IS), where *p* denotes the number of markers.

Step 4. Each row of the standardized matrix of markers ( M) is then multiplied by the vector of weights (**w**), in matrix notation we build the diagonal matrix of squared weights, as **D =** Diag**(**$w_{1}^{2},w_{2}^{2},\ldots ,w_{p}^{2}$**)**, and finally, with this weighted matrix, a weighted GRM is computed as $G^{*}=\frac{MDM^{T}}{p}$. The implementation of model (1) using the weighted GRM $\left(G^{*}\right)$ is what we call method AB. It is important to point out that the GBLUP and AB methods were implemented in the R statistical software (R Core Team 2023).

The AB method works primarily from its capability to minimize the mismatch between the training and testing distributions. This is achieved by diminishing the weight assigned to markers that exhibit the most pronounced differences between the training and testing sets. In simple words, the weights resulting from the AB method originate from a binary classification model and reflect the significance of each variable (marker) in distinguishing between training and testing sets. By assigning a value of one to observations belonging to the testing set and zero to those from the training set, the weights indicate the relative importance of independent variables (markers) in this differentiation process. In essence, higher importance scores associated with certain variables imply a more substantial contribution to the ability to differentiate observations between the training and testing sets. Also, it is important to point out that the proposed AB method is not restricted only to family prediction, since it can be used for any type of prediction where there is a significant mismatch between the training and testing distributions.

### Data

Details of the 12 data sets are shown in Table A1 (Appendix).

### Evaluation of prediction accuracy

The cross-validation approach used in this study involved leaving one family out. In each iteration, the data from a single-family served as the testing set, while the data from all other families constituted the training set (Montesinos-López *et al*. 2022). The number of iterations was equal to the number of families to ensure that each family was used as the testing set exactly one time. This method was employed to assess the model's ability to predict information from a complete family using data from different and diverse families.

To assess the predictive efficacy of the proposed AB method in contrast to the GBLUP model, three metrics were employed. Initially, the mean square error (MSE) was utilized to gauge the model's prediction accuracy by measuring the squared deviation between observed and predicted values on the testing set. Subsequently, the average Pearson's correlation (COR) was calculated to ascertain the strength and direction of the linear relationship between the observed and predicted values on the testing set. Additionally, the normalized root mean square error (NRMSE), serving as another metric for prediction error, was computed. To derive this metric, we first calculated the square root of the MSE and then divided this value by the average of the observed values in the testing set. These evaluation metrics provided valuable insights into the performance of the proposed AB method relative to the GBLUP method in predicting information across an entire family.

In addition, we computed the relative efficiency (RE), in terms of MSE, NRMSE, and COR of each model with the following expressions:


$$RE_{MSE}=\left(\frac{MSE(M_{GBLUP})}{MSE(M_{AB})}\right)\;$$

$$RE_{NRMSE}=\left(\frac{NRMSE(M_{GBLUP})}{NRMSE(M_{AB})}\right)\;$$

where $M_{GBLUP}$ and $M_{AB}\;$ denote the models compared, the GBLUP and the AB model. The RE for COR was computed as:


$$RE_{COR}=\left(\frac{COR(M_{AB})}{COR(M_{GBLUP})}\right)\;$$

Across the three measures of relative efficiency (RE_MSE, RE_NRMSE, and RE_COR), a value greater than one signifies a superior performance of the AB method. Conversely, a value <1 indicate the GBLUP method's superiority. A value equal to 1 suggests equivalent performance between the GBLUP and AB methods.

## Results

**Fig. 1.**
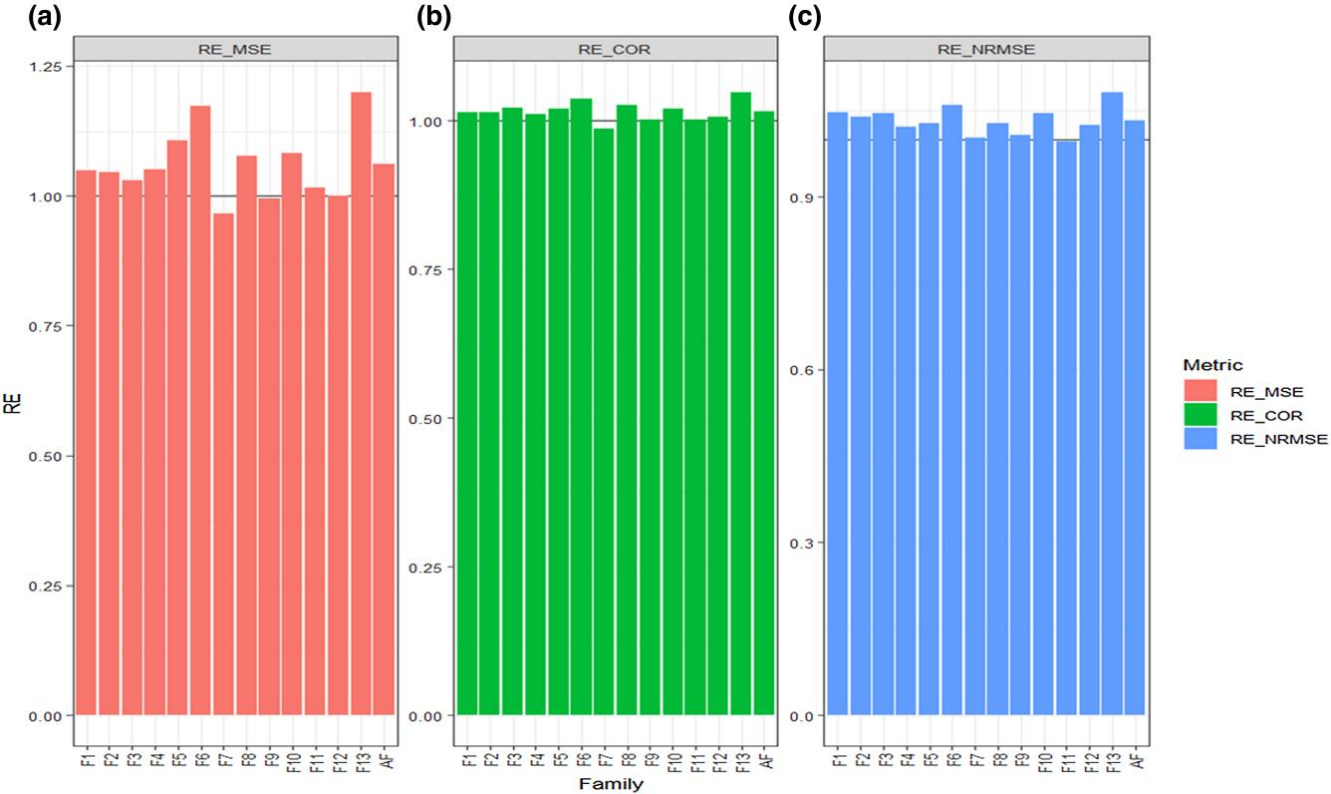
Data 1 (GDM). Relative efficiency (RE) between the proposed method (AB with Boruta for variable selection) and the GBLUP method for each family and across family (AF) in terms of a) mean square error, MSE; b) average Pearson’s correlation, COR; and c) normalized root mean square error, NRMSE. RE > 1 means that the AB method outperformed the GBLUP method, RE < 1 means that the GBLUP method outperformed the AB method and RE = 1 means that both methods performed equally.

**Fig. 2.**
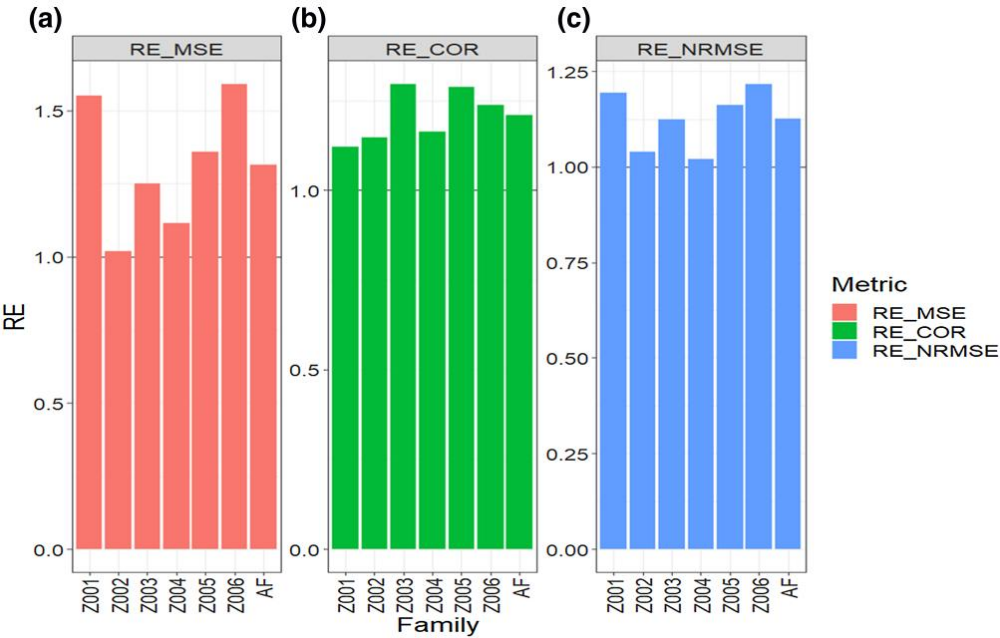
Data 2 (Maize_1). Relative efficiency (RE) between the proposed method (AB with Boruta for variable selection) and the GBLUP method for each family and across family (AF) in terms of a) mean square error, MSE; b) average Pearson’s correlation, COR; and c) normalized root mean square error, NRMSE. RE > 1 means that the AB method outperformed the GBLUP method, RE < 1 means that the GBLUP method outperformed the AB method and RE = 1 means that both methods performed equally.

**Fig. 3.**
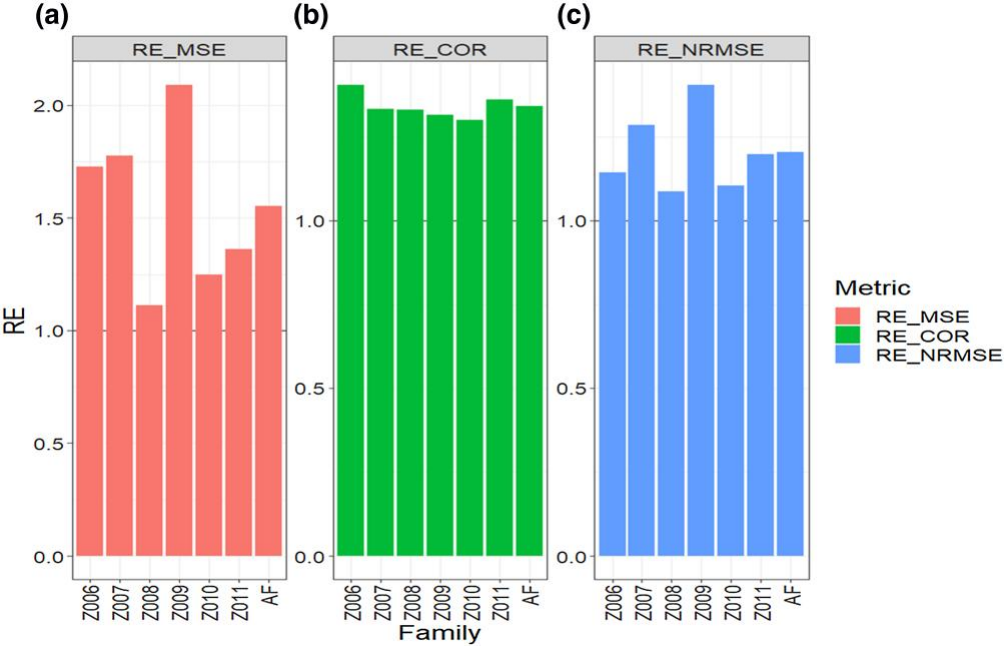
Data 3 (Maize_2). Relative efficiency (RE) between the proposed method (AB with Boruta for variable selection) and the GBLUP method for each family and across family (AF) in terms of a) mean square error, MSE; b) average Pearson’s correlation, COR; and c) normalized root mean square error, NRMSE. RE > 1 means that the AB method outperformed the GBLUP method, RE < 1 means that the GBLUP method outperformed the AB method and RE = 1 means that both methods performed equally.

**Fig. 4.**
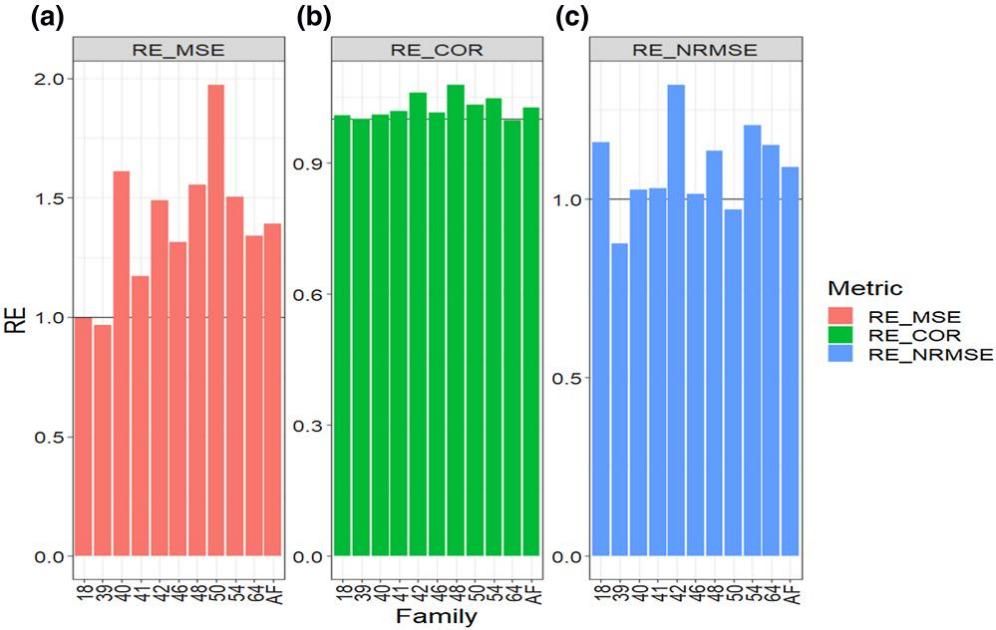
Data 9 (Soybean_4). Relative efficiency (RE) between the proposed method (AB with Boruta for variable selection) and the GBLUP method for each family and across family (AF) in terms of a) mean square error, MSE; b) average Pearson’s correlation, COR; and c) normalized root mean square error, NRMSE. RE > 1 means that the AB method outperformed the GBLUP method, RE < 1 means that the GBLUP method outperformed the AB method and RE = 1 means that both methods performed equally.

**Fig. 5.**
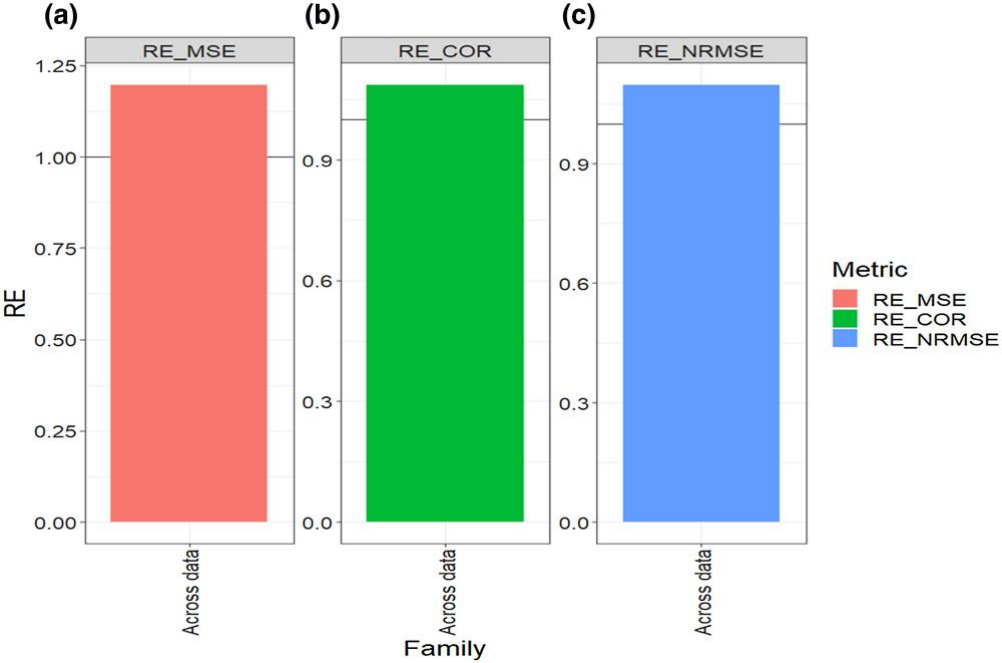
Across datasets. Relative efficiency (RE) between the proposed method (AB with Boruta for variable selection) and the GBLUP method for each family and across family (AF) in terms of a) mean square error, MSE; b) average Pearson’s correlation, COR; and c) normalized root mean square error, NRMSE. RE > 1 means that the AB method outperformed the GBLUP method, RE < 1 means that the GBLUP method outperformed the AB method and RE = 1 means that both methods performed equally.

The results are organized into 5 sections. The 4 first sections present the results for Data 1 (GDM), Data 2 (Maize_1), Data 3 (Maize 2), and Data 9 (Soybean_4), while section 5 presents the results across all data sets under study. For each data set, we compared the results between the GBLUP and the proposed AB method in terms of MSE, COR, and NRMSE. The results for Data 1–3 and Data 9 and across data sets are shown in Figs. 1–5 and Tables 1–5, respectively.

**Table 1.** Data 1 (GDM).

Data_set	Family	MSE_GBLUP	COR_GBLUP	NRMSE_GBLUP	MSE_AB	COR_AB	NRMSE_AB	RE_MSE	RE_COR	RE_NRMSE
GDM	F1	119,918.048	0.812	0.511	114,336.072	0.824	0.488	1.049	1.014	1.046
GDM	F2	132,406.015	0.840	0.515	126,595.797	0.851	0.496	1.046	1.014	1.039
GDM	F3	128,531.101	0.751	0.524	124,833.302	0.768	0.501	1.030	1.022	1.045
GDM	F4	124,508.028	0.837	0.473	118,403.764	0.846	0.463	1.052	1.011	1.021
GDM	F5	110,073.458	0.859	0.444	99,472.574	0.877	0.432	1.107	1.021	1.028
GDM	F6	206,341.443	0.801	0.543	175,968.963	0.831	0.513	1.173	1.037	1.059
GDM	F7	124,698.294	0.747	0.601	129,174.949	0.737	0.600	0.965	0.986	1.002
GDM	F8	152,335.628	0.760	0.600	141,327.354	0.780	0.584	1.078	1.026	1.028
GDM	F9	137,713.335	0.870	0.437	138,444.200	0.872	0.434	0.995	1.002	1.007
GDM	F10	121,695.210	0.822	0.483	112,492.040	0.839	0.462	1.082	1.020	1.045
GDM	F11	119,274.742	0.833	0.558	117,417.465	0.834	0.560	1.016	1.001	0.996
GDM	F12	132,365.930	0.863	0.470	132,251.696	0.867	0.459	1.001	1.006	1.025
GDM	F13	108,448.837	0.789	0.509	90,377.859	0.826	0.470	1.200	1.048	1.082
GDM	AF	132,177.698	0.814	0.513	124,699.695	0.827	0.497	1.061	1.016	1.033

Prediction accuracy in terms of mean square error (MSE), average Pearson’s correlation (COR) and Normalized mean square error (NRMSE). The metrics that end with_GBLUP denote the results under the GBLUP, while those that end with _AB denote the AB method with variable selection using the Boruta algorithm. Relative efficiency (RE) denotes the RE for each metrics, RE_MSE and RE_NRMSE were computed dividing the MSE_GBLUP by the MSE_AB, and the NRMSE_GBLUP by the NRMSE_AB, while the RE_COR was computed dividing the COR_AB by the COR_GBLUP. RE values larger than one indicate that the AB method outperformed the GBLUP method.

Supplementary Material contains results for the rest of the data sets, Data 4–8 (Maize_3, Maize_4, Soybean_1, Soybean_2, Soybean_3, respectively) and Data 10–12 (Maize_Bi_2018, Maize_Bi_2019_D, and Maize_Bi_2019_O, respectively), are presented in Supplementary Tables 1–8, respectively, and displayed in Supplementary Figs. 1–8, respectively.

Note that for simplicity purposes, Supplementary Materials only show the description of results from Data 4–8 and of Data set 10 (Maize_3, Maize_4, Soybean_1, Soybean_2, Soybean_3, and Maize_Bi_2018, respectively) given in Supplementary Tables 1-6 and Supplementary Figs. 1-6. Although results from data sets Data 11 to 12 (Maize_Bi_2019_D and Maize_Bi_2019_O, respectively) are shown in Supplementary Tables 7–8 and Supplementary Figs. 7-8, respectively, they are not discussed in the text of Supplementary Material.

### Data 1 (GDM data set)

In this section, we compared the GBLUP and AB methods for each family across traits for the “GDM” dataset. The comparison was done using three metrics (MSE, COR, and NRMSE) and for each metric, the RE was computed, comparing the GBLUP and AB methods (Fig. 1 and Table 1).

In terms of MSE we found that in 11 out of the 13 families the RE > 1, this means that the AB method outperformed the GBLUP method in 11 out of the 13 families. However, across traits and families we got an RE = 1.061, meaning that, on average, the AB method outperformed the GBLUP method by 6.1% in terms of MSE (Table 1 and Fig. 1).

Furthermore, in terms of COR, we found that the AB method was better than the GBLUP method in 12 out of 13 families, since in 12 of these families, the RE, in terms of COR, was greater than 1. Meanwhile, across traits and families, the gain of the AB method regarding the GBLUP method was 1.6% since the RE = 1.016 (Fig. 1, Table 1).

Finally, in terms of NRMSE values, Fig. 1 and Table 1 show that 12 out of 13 of the RE values are larger than 1, which means that the AB method outperformed the GBLUP method in 12 out of 13 families. Across traits and families, we found an RE = 1.033, which means that the AB method gains the GBLUP method by 3.3% in terms of prediction performance. In sum, across traits, family and metrics method AB outperforms method GBLUP in this Data 1.

### Data 2 (Maize_1 data set)

In this section, we conducted a comparative analysis of the GBLUP and AB methods across traits for Data 2. The same three metrics (MSE, COR, and NRMSE) were used to measure the performance of both methods. The results are presented in Fig. 2 and Table 2.

**Table 2.** Data 2 (Maize_1).

Data_set	Family	MSE_GBLUP	COR_GBLUP	NRMSE_GBLUP	MSE_AB	COR_AB	NRMSE_AB	RE_MSE	RE_COR	RE_NRMSE
Maize_1	Z001	6551.470	0.529	1.255	4220.248	0.593	1.051	1.552	1.121	1.194
Maize_1	Z002	1748.914	0.547	0.890	1715.049	0.627	0.857	1.020	1.147	1.038
Maize_1	Z003	2294.720	0.480	0.919	1834.473	0.622	0.817	1.251	1.296	1.124
Maize_1	Z004	2078.756	0.547	0.908	1865.140	0.636	0.889	1.115	1.162	1.021
Maize_1	Z005	2689.596	0.495	0.931	1978.842	0.637	0.801	1.359	1.287	1.162
Maize_1	Z006	2608.669	0.533	1.066	1638.615	0.659	0.876	1.592	1.237	1.217
Maize_1	AF	2995.354	0.522	0.995	2208.728	0.629	0.882	1.315	1.208	1.126

Prediction accuracy in terms of mean square error (MSE), average Pearson’s correlation (COR) and Normalized mean square error (NRMSE). The metrics that end with_GBLUP denote the results under the GBLUP, while those that end with _AB denote the AB method with variable selection using the Boruta algorithm.Relative efficiency (RE) denotes the relative efficiency for each metric, RE_MSE and RE_NRMSE were computed dividing the MSE_GBLUP by the MSE_AB, and the NRMSE_GBLUP by the NRMSE_AB, while the RE_COR was computed dividing the COR_AB by the COR_GBLUP. RE values larger than one indicate that the AB method outperformed the GBLUP method.

Regarding MSE, we observed that the AB method outperformed the GBLUP method in all six families, with RE values >1. This indicates that the AB method displayed superior predictive capability in all instances. Across all traits and families, the overall RE of the AB method was found to be 1.315, representing an average improvement of 31.5% over the GBLUP method in terms of MSE (Table 2 and Fig. 2).

Similarly, in terms of COR, the AB method displayed a better performance than the GBLUP method in all six families, as evidenced by RE values >1 for each family. The overall gain of the AB method relative to the GBLUP method, considering all traits and families, was 20.8%, with an RE value of 1.208 (Fig. 2 and Table 2).

Additionally, when considering NRMSE values, we again observed that the AB method outperformed the GBLUP method in all 6 families, with RE values consistently above 1. The overall RE across traits and families was 1.126, indicating a 12.6% improvement in prediction performance for the AB method compared to the GBLUP method.

### Data 3 (Maize_2 data set)

In this section, we conducted a comprehensive comparison between the GBLUP and AB methods across traits for the Maize_2 dataset with the same metrics. Regarding MSE, our analysis revealed that the AB method outperformed the GBLUP method in all six families, as evidenced by RE values >1. This indicates a consistent superiority of the AB method over the GBLUP method within each family. When considering all traits and families together, the overall RE value was 1.553, indicating an average improvement of 55.3% in favor of the AB method in terms of MSE (Table 3 and Fig. 3).

**Table 3.** Data 3 (Maize_2).

Data_set	Family	MSE_GBLUP	COR_GBLUP	NRMSE_GBLUP	MSE_AB	COR_AB	NRMSE_AB	RE_MSE	RE_COR	RE_NRMSE
Maize_2	Z006	2311.286	0.471	1.071	1338.993	0.661	0.937	1.726	1.404	1.143
Maize_2	Z007	2523.508	0.501	1.032	1421.342	0.667	0.804	1.775	1.331	1.284
Maize_2	Z008	4866.393	0.479	1.205	4367.755	0.636	1.110	1.114	1.329	1.086
Maize_2	Z009	3301.102	0.480	1.159	1578.735	0.631	0.825	2.091	1.314	1.404
Maize_2	Z010	3216.048	0.479	1.071	2572.810	0.622	0.971	1.250	1.298	1.103
Maize_2	Z011	7317.351	0.408	1.511	5371.310	0.554	1.263	1.362	1.359	1.196
Maize_2	AF	3922.615	0.470	1.175	2775.157	0.629	0.985	1.553	1.339	1.203

Prediction accuracy in terms of mean square error (MSE), average Pearson’s correlation (COR), and normalized mean square error (NRMSE). The metrics that end with_GBLUP denote the results under the GBLUP, while those that end with _AB denote the AB method with variable selection using the Boruta algorithm. Relative efficiency (RE) denotes the RE for each metric, RE_MSE and RE_NRMSE were computed dividing the MSE_GBLUP by the MSE_AB, and the NRMSE_GBLUP by the NRMSE_AB, while the RE_COR was computed dividing the COR_AB by the COR_GBLUP. RE values larger than one indicate that the AB method outperformed the GBLUP method.

Furthermore, in terms of COR, the AB method displayed a higher performance compared to the GBLUP method in all 6 families, as indicated by RE values larger than 1 for each family. Across all traits and families, the AB method achieved an RE value of 1.339, representing a 33.9% gain over the GBLUP method (Fig. 3 and Table 3).

Similarly, the NRMSE values exhibited a consistent pattern of improvement for the AB method. In all six families, the AB method outperformed the GBLUP method, with RE values surpassing 1. When considering all traits and families collectively, the overall RE value was 1.203, indicating a 20.3% performance gain of the AB method over the GBLUP method.

### Data 4 (Soybean_4 dataset)

Regarding the MSE metric, our findings indicate that the AB method outperformed the GBLUP method in 8 out of the 10 families, with an RE value >1. Across all traits and families, the average improvement achieved by the AB method vs the GBLUP method was 39.2% (RE = 1.392, Table 4 and Fig. 4).

**Table 4.** Data 9 (Soybean_4).

Data_set	Family	MSE_GBLUP	COR_GBLUP	NRMSE_GBLUP	MSE_AB	COR_AB	NRMSE_AB	RE_MSE	RE_COR	RE_NRMSE
Soybean_4	18	68,070.522	0.815	0.704	68,317.303	0.823	0.607	0.996	1.009	1.160
Soybean_4	39	143,007.765	0.831	0.556	148,089.245	0.831	0.636	0.966	1.000	0.875
Soybean_4	40	74,413.189	0.856	0.622	46,240.386	0.865	0.606	1.609	1.011	1.026
Soybean_4	41	111,374.677	0.809	0.544	94,994.783	0.824	0.527	1.172	1.018	1.031
Soybean_4	42	182,961.181	0.754	0.747	122,794.534	0.800	0.566	1.490	1.060	1.321
Soybean_4	46	96,267.088	0.789	0.598	73,260.793	0.800	0.590	1.314	1.014	1.015
Soybean_4	48	82,289.105	0.723	0.681	52,981.294	0.780	0.600	1.553	1.079	1.136
Soybean_4	50	162,327.393	0.779	0.601	82,244.828	0.804	0.618	1.974	1.033	0.972
Soybean_4	54	150,133.279	0.782	0.680	99,797.457	0.818	0.563	1.504	1.047	1.207
Soybean_4	64	315,164.344	0.869	0.882	235,267.795	0.867	0.765	1.340	0.997	1.152
Soybean_4	AF	138,600.854	0.801	0.662	102,398.842	0.821	0.608	1.392	1.027	1.090

Prediction accuracy in terms of mean square error (MSE), average Pearson’s correlation (COR), and normalized mean square error (NRMSE). The metrics that end with_GBLUP denotes the results under the GBLUP, while those that end with _AB denotes the AV method with variable selection using the Boruta algorithm. Relative efficiency (RE) denotes the RE for each metric, RE_MSE and RE_NRMSE were computed dividing the MSE_GBLUP by the MSE_AB, and the NRMSE_GBLUP by the NRMSE_AB, while the RE_COR was computed dividing the COR_AB by the COR_GBLUP. RE values larger than one indicate that the AB method outperformed the GBLUP method.

Similarly, regarding the COR metric, the AB method displayed a higher performance in 9 out of 10 families, as indicated by RE >1. Across all traits and families, the AB method showed a modest gain of 2.7% compared to the GBLUP method (RE = 1.027, Fig. 4, Table 4).

Additionally, the NRMSE metric analysis revealed that in 8 out of 10 cases, the RE values exceeded 1, highlighting the advantage of the AB method over the GBLUP method. Overall, across all traits and families, the AB method displayed a 9.0% improvement in prediction performance, as evidenced by an RE value of 1.090.

### Across data sets

Regarding the MSE metric, our findings across data sets and families indicate that the AB method outperformed the GBLUP method by 19.7% (RE = 1.19.7, Table 5 and Fig. 5). Turning our attention to the COR metric, the AB method displayed a higher performance with a gain of 8.6% compared to the GBLUP method (RE = 1.086, Fig. 5, Table 5). Additionally, in terms of NRMSE, the AB method exhibited an improvement of 9.8% over the GBLUP method, as indicated by a RE value of 1.098 (as depicted in Fig. 5 and Table 5).

**Table 5.** Across datasets.

Data_set	Family	MSE_GBLUP	COR_GBLUP	NRMSE_GBLUP	MSE_AB	COR_AB	NRMSE_AB	RE_MSE	RE_COR	RE_NRMSE
Across data sets	Across data	42,695.770	0.535	4.864	37,885.753	0.585	4.415	1.197	1.086	1.098

Prediction accuracy in terms of mean square error (MSE), average Pearson’s correlation (COR), and normalized mean square error (NRMSE). The metrics that end with_GBLUP denote the results under the GBLUP, while those that end with _AB denote the AV method with variable selection using the Boruta algorithm. Relative efficiency (RE) denotes the RE for each metric, RE_MSE and RE_NRMSE were computed dividing the MSE_GBLUP by the MSE_AB, and the NRMSE_GBLUP by the NRMSE_AB, while the RE_COR was computed dividing the COR_AB by the COR_GBLUP. RE values larger than one indicate that the AB method outperformed the GBLUP method.

In sum, our findings consistently found AB method superior to the GBLUP method across diverse datasets, traits and family categories. These results indicate that the AB method is indeed effective in predicting untested families when trained on tested ones.

## Discussion

Family prediction in breeding programs is of paramount importance, since it is essential to optimize the use of resources, accelerate the breeding cycle, reduce environmental impact, and produce new plant varieties with improved traits. Family prediction allows breeders to identify and select superior genotypes based on their genetic potential. By predicting the performance of entire families rather than individual plants, breeders can make informed decisions about which families are likely to exhibit desirable traits such as higher yield, resistance to diseases, and improved quality.

Family prediction is very important since enables breeders to allocate their resources more efficiently by focusing on families with the highest predicted performance. This targeted approach accelerates the breeding process, allowing for the identification of promising plant varieties more quickly. As a result, resources such as time, manpower, and field space are utilized more effectively, leading to the development of improved crop varieties in a timelier manner. This efficiency is crucial in addressing global challenges such as food security, climate change, and evolving pest and disease pressures.

Challenging family predictions in plant breeding can arise due to several factors, which can complicate the process and impact the accuracy of predictions. Some of these challenges include (1) genetic variation within families, (2) quantitative traits and polygenicity, (3) epistasis and gene interactions, (4) recombination and linkage disequilibrium, (5) limited sample size, etc. Addressing these challenges requires a combination of advanced statistical methods, cutting-edge genomic technologies, robust experimental designs, careful consideration of environmental factors, and iterative model refinement. Although genomics and statistical modeling continue to improve the accuracy of family predictions in plant breeding, it is likely that these challenges will continue to be important.

For this reason, in this research, a novel method was proposed that integrates the strengths of the AV method and Boruta method and it is called the AB method. This method trains binary classifiers for each family with a fictitious response variable. The fictitious response variable is labeled as 1 (testing set) when the input belongs to the family of interest, while the inputs of the remaining families are labeled as 0 (training set). During the training process with the Boruta method, each original marker and its permuted (shuffled) version is used as input. This process helps determine marker importance. Original markers are then weighted using the inverse of their computed scores, and with these weighted markers, the weighted GRM is computed. The GBLUP model is then implemented using this weighted GRM. From our results we speculate that the AB method is simply picking up the SNPs segregating on the test set. In which case, a quality control process on the SNP set could be used to tailor the model to specific families. The proposed AB method was compared to the GBLUP model that uses a GRM without weights on 12 real datasets.

We found the AB method outperformed the GBLUP method between 0% (Data 12, Maize_Bi_2019_O) and 55.3% (Data 3, Maize_2) in terms of MSE, between 0% (Data 12, Maize_Bi_2019_O) and 33.9% (Data 3, set Maize_2) in terms of COR and between 1.1% in Data 12, Maize_Bi_2019_O) and 33.9% (Data 3, Maize_2) in terms of NRMSE. On average across data sets, traits and families, we observed that the AB method outperformed the GBLUP method by 8.6, 19.7, and 9.8% in terms of COR, MSE and normalized root MSE. These results are very promising, since they provide empirical evidence that the proposed AB method can help to significantly increase the prediction performance of complete families.

The power of the AB method mostly is attributed to the fact that it reduces the mismatch between the training and testing distribution by reducing the weight of those markers that most differentiate the training from the testing set. This adjustment is possible to be carried out due to the hybrid nature of the AB method that uses the AV approach proposed by Montesinos-López, Kismiantini *et al*. (2023) for the context of plant breeding to quantify the magnitude of the mismatch between the training and testing sets, followed by the Boruta method to identify those markers that are more important in the differentiation between the training and testing set. Then with the inverse of the importance scores a weighted GRM is built that is used coupled with the GBLUP model with the goal of improving the prediction performance. Our results are encouraging since we not only reduced prediction error (with MSE and NRMSE) but also increases predictive ability (as Pearson’s correlation between predicted and observed values) which is one of the most popular metrics used in GS, and that guarantees a better selection process of the best (top or bottom) candidate genotypes. Also, it is important to point out that the proposed AB method is not restricted to only hybrid predictions since this method can be applied to any prediction problem in which exist a significant mismatch between the training and testing set, also the size of the training and testing set have not restrictions, that is, the percentages of observations in the training and testing can be quite different.

However, even though the results are very promising, in some data sets we did not find a significant improvement in the prediction accuracy of some families, which in part can be attributed to a not significant mismatch between the training and testing sets and in other to difficulties of building robust statistical machine learning methods for complete family predictions since, as mentioned above, many factors that interact in complex ways affect the family performance. Also, it is hard to predict some families because the heritability for a single family within a single environment is very low, unless this family happens to be segregating to a major QTL and in these cases, the test is too noisy to validate the methodology.

For example, the number of individuals per family (family size) is key to improving the prediction accuracy of a complete family, since the larger the family size, we will have more information to train the genomic prediction model efficiently. In addition, it is key to have many families in the training set since, in this way, the probability that the family to be predicted has more similar families in the training set increases.

Also, the size and structure of the training population affect the accuracy of genomic prediction models (VanRaden *et al*. 2009; Daetwyler *et al*. 2010; Habier *et al*. 2010; de Bem Oliveira *et al*. 2020). Furthermore, incorporating more than 10 individuals within each family will diminish the sampling variability of both allele frequency and phenotypic mean, ultimately leading to enhanced genomic accuracies (de Bem Oliveira *et al*. 2020). However, we are aware that not even the family can predict itself well because of factors such as G × E (Alencar 2021).

For the situation mentioned above, it is crucial to highlight the significance of genome-wide family prediction in breeding practices, particularly considering that numerous species are cultivated within large full or half-sibling family populations, often serving as commercially viable populations with varying degrees of relatedness, as seen in certain forage species such as alfalfa (*Medicago sativa* L.) (Annicchiarico *et al.* 2015; Biazzi *et al*. 2017) and ryegrass (*Lolium perenne* L.) (Fè *et al*. 2016). Within these species, the family unit, whether composed of full or half-siblings, constitutes the fundamental entity for phenotyping, typically performed at the plot level to measure attributes like yield, as opposed to the individual plant level. This approach is required by the allogamous nature of their mating systems (Poehlman 1987). Notably, individual plants hold limited significance, given that commercial varieties are essentially homogeneous populations comprising heterozygous individuals (Poehlman 1987).

Significantly, the application of genome-wide family prediction has already been reported in crops that are intentionally bred and cultivated as family pools, particularly in cross-pollinated forage species (Fè *et al.* 2016; Annicchiarico *et al.* 2015; Biazzi *et al*. 2017). For this reason, the proposed AB method is a promising tool that helps to improve the prediction performance of complete families, but still further research is required to be able to improve the modeling process in family predictions in such a way that, it is highly probable that we can guarantee a high prediction accuracy of each family, in any trait and in any data set. The application of the AB method generally improves the prediction accuracy or, in case this does not occur, the genomic prediction accuracy will not be negatively affected by the application of the AB method.

## Conclusions

Due to the need to improve family prediction accuracy in plant breeding programs, we proposed the AB method that integrates the AV method with the Boruta method. The former detects the presence and magnitude of the mismatch between the training and testing set using a binary classifier using the original features (inputs) and a fictitious response variable, while the Boruta computes feature importance, also using the same fictitious response variable, then with the inverse of the feature importance scores the original features (markers) are weighted, and using them, a weighed GRM is computed. Finally, the GBLUP model is used with the weighted GRM. We found that the proposed AB method outperforms the GBLUP by 8.6, 19.7, and 9.8% in terms of Pearson’s correlation, MSE, and normalized root MSE across data sets, traits, and families. The proposed AB method was shown to be efficient in most data sets under study, but for cases in the AB method did not produce any increase in genomic prediction accuracy, the AB method did not produce any decrease in the accuracy of the prediction. Certainly, more empirical evaluations are welcome to support our findings.

## Supplementary Material

jkad278_Supplementary_Data

## Data Availability

The data sets used in this study can be downloaded from the following link https://hdl.handle.net/11529/10548950. While the R code is available at https://github.com/osval78/Adversarial_Boruta_-AB-_Method. Supplemental material available at G3 online.

## Appendix

**Table A1.** Detailed description of the data sets used to implement the proposed AB method for parental selection.

Data number	Data set	Genotypes	Markers	Environments	Traits	Families	Trait name
Data 1	GDM Seed Soybean	532	626	12	2	13	Grain yield and moisture
Data 2	Maize_1	1000	4085	11	4	6	Days to tassel, anthesis silking interval, plant height, and ear height
Data 3	Maize_2	1000	4085	11	4	6	Days to tassel, anthesis silking interval, plant height, and ear height
Data 4	Maize_3	1000	4085	11	4	6	Days to tassel, anthesis silking interval, plant height, and ear height
Data 5	Maize_4	999	4085	11	4	6	Days to tassel, anthesis silking interval, plant height, and ear height
Data 6	Soybean_1	1044	1810	8	6	8	Plant height, R8, planting, maturity, lodging, and grain yield
Data 7	Soybean_2	691	1808	8	6	8	Plant height, R8, planting, maturity, lodging, and grain yield
Data 8	Soybean_3	70	1809	8	6	10	Plant height, R8, planting, maturity, lodging, and grain yield
Data 9	Soybean_4	59	1803	8	6	10	Plant height, R8, planting, maturity, lodging, and grain yield
Data 10	Maize_Bi_2018	1284	5465	1	21	29	Grain yield, GYgrn, AD, SD, ASI, EH, PH, rEPH, rEPP, EarAsp, MOI, PltAsp, SAT, nP, pER, pRL, pSL, Eturc1, GLS, MSV, and pBHC
Data 11	Maize_Bi_2019_D	722	8754	1	16	19	Grain yield, GYgrn, AD, ASI, EH, EarAsp, MOI, PH, SAT, SD, nP, pER, pRL, pSL, rEPH, and rEPP
Data 12	Maize_Bi_2019_O	722	8754	1	20	19	Grain yield, GYgrn, AD, ASI, EH, EarAsp, MOI, PH, SAT, SD, nP, pER, pRL, pSL, rEPH, rEPP, Eturc1, GLS, PltAsp, and pBHC
