# Supplementary material for: A marker weighting approach for enhancing within-family accuracy in genomic prediction
Source: G3 (Bethesda). 2023 Dec 11;14(2):jkad278. doi: 10.1093/g3journal/jkad278 (PMC10849334; doi:10.1093/g3journal/jkad278)
Supplement: jkad278_Supplementary_Data [file jkad278_supplementary_data.docx]

**SUPPLEMENTARY MATERIAL**

**Data 4** (Maize_3)

In this section, we compared the GBLUP and AB methods for each family across traits for the "Maize_3" dataset. The comparison was carried out using three metrics (MSE, COR and NRMSE) and for each metric, the RE efficiency was computed comparing the GBLUP and AB methods (**Figure C1** and **Table B1**).

In terms of MSE, we found that in 5 out of the 6 families the RE>1, this means that the AB method outperformed the C method in 5 out of the 6 families. However, across traits and family we got an RE=1.349, meaning that, on average, the AB method outperformed the GBLUP method by 34.9% in terms of MSE (**Table B1** and **Figure C1**).

Furthermore, in terms of COR, we found that the AB method was better than the G method in 6 out of 6 families, since in all families the RE, in terms of COR, was larger than 1. Meanwhile, across traits and families, the gain of the AB method regarding the C method was of 16.9%, since the RE=1.169 (**Figure C1,** **Table B1**).

Finally, in terms of NRMSE, values also can be observed in Figure C1 and Table B.1 that in 6 out 6 of the RE values are larger than 1, which means that the AB method outperformed the C method in all families (6). Across traits and families, we found an RE=1.156, which means that the AB method gains the C method by 15.6% in terms of prediction performance. In conclusion, for all traits, families and metrics, the AB method strongly outperforms the GBLUP method in this Data set 4. These results indicate that the AB method is more effective when predicting untested families using tested families as training.

**Data 5** (Maize_4)

In this section, we carried out a comprehensive comparison between the GBLUP and AB methods across different traits for the "Maize_4" dataset. The evaluation involved three key metrics: Mean Squared Error (MSE), Correlation (COR), and Normalized Root Mean Squared Error (NRMSE). For each metric, we computed the relative efficiency (RE) to compare the performance of the GBLUP and AB methods (**Figure C2** and **Table B2**).

Regarding the MSE metric, our findings indicate that the AB method outperformed the GBLUP method in all 6 families, as evidenced by RE values greater than 1 per family. When considering all traits and families, the average improvement achieved by the AB method was 52.0% (RE = 1.520, **Table B2** and **Figure C2**).

Furthermore, regarding the COR metric, the AB method displayed a higher performance in all 6 families, as all families showed RE values greater than 1. Across all traits and families, the AB method showcased a substantial gain of 21.3% compared to the GBLUP method (RE = 1.213, **Figure C2**, **Table B2**).

Additionally, the analysis of NRMSE values revealed that the AB method consistently outperformed the GBLUP method in all 6 families, with RE values greater than 1 for each family. Overall, across all traits and families, the AB method demonstrated a significant improvement of 16.8% in prediction performance, as indicated by an RE value of 1.168 (**Figure C2**, **Table B2**).

In conclusion, across all traits, families, and metrics, the AB method demonstrated a strong superiority over the GBLUP method in Data 5 (Maize_4). These results indicate that the AB method is more effective in predicting untested families using tested families as training data.

**Data 6** (Soybean_1)

In this section, we conducted a comprehensive comparison between the GBLUP and AB methods across different traits for the "Soybean_1" dataset. The evaluation involved three key metrics: Mean Squared Error (MSE), Correlation (COR), and Normalized Root Mean Squared Error (NRMSE). For each metric, we computed the relative efficiency (RE) to compare the performance of the GBLUP and AB methods (**Figure C3** and **Table B3**).

Regarding the MSE metric, our findings indicate that the AB method outperformed the GBLUP method in 8 out of the 10 families, as evidenced by RE values greater than 1 for these families. Across all traits and families, the AB method exhibited an average improvement of 3.5% over the GBLUP method (RE = 1.035, **Table B3** and **Figure C3**).

Furthermore, regarding the COR metric, the AB method demonstrated superior performance in all 10 families, as indicated by RE values greater than 1 for each family. Across all traits and families, the AB method showcased a notable gain of 4.6% compared to the GBLUP method (RE = 1.046, **Figure C3**, **Table B3**).

Additionally, the analysis of NRMSE values revealed that the AB method outperformed the GBLUP method in 7 out of the 10 families, with RE values greater than 1 for these cases. Overall, across all traits and families, the AB method demonstrated a significant improvement of 8.3% in prediction performance, as indicated by an RE value of 1.083 (**Figure C3, Table B3**).

In conclusion, the results consistently demonstrate that the AB method outperforms the GBLUP method across various traits, families, and metrics in Data 6 (Soybean_1). These findings indicate that method AB is slightly more effective in predicting untested families using tested families as training data.

**Data 7** (Soybean_2)

In this section, we conducted a comprehensive comparison between the GBLUP and AB methods across different traits for the "Soybean_2" dataset. The evaluation involved three key metrics: Mean Squared Error (MSE), Correlation (COR), and Normalized Root Mean Squared Error (NRMSE). For each metric, we computed the relative efficiency (RE) to compare the performance of the GBLUP and AB methods (**Figure C4** and **Table B4**).

Regarding the MSE metric, our findings indicate that the AB method outperformed the GBLUP method in 5 out of the 10 families, as evidenced by RE values greater than 1 for these families. Across all traits and families, the AB method exhibited an average improvement of 4.7% over the GBLUP method (RE = 1.047**, Table B4** and **Figure C4**).

Furthermore, regarding the COR metric, the AB method demonstrated superior performance in all 10 families, as indicated by RE values greater than 1 for each family. Across all traits and families, the AB method showcased a notable gain of 10.2% compared to the GBLUP method (RE = 1.102, **Figure C4**, **Table B4**).

Additionally, the analysis of NRMSE values revealed that the AB method outperformed the GBLUP method in 9 out of the 10 families, with RE values greater than 1 for these cases. Overall, across all traits and families, the AB method demonstrated a significant improvement of 16.1% in prediction performance, as indicated by an RE value of 1.161 (**Figure C4**, **Table B4**).

In conclusion, the results consistently demonstrate that the AB method outperforms the GBLUP method across various traits, families, and metrics in Data 7. These findings indicate that method AB is slightly more effective in predicting untested families using tested families as training data.

**Data 8** (Soybean_3)

In this section, we conducted a comprehensive comparison between the GBLUP and AB methods across different traits for the "Soybean_3" dataset. The evaluation involved three key metrics: Mean Squared Error (MSE), Correlation (COR), and Normalized Root Mean Squared Error (NRMSE). For each metric, we computed the relative efficiency (RE) to compare the performance of the GBLUP and AB methods (**Figure C5** and **Table B5**).

Regarding the MSE metric, our findings indicate that the AB method outperformed the GBLUP method in 5 out of the 10 families, as evidenced by RE values greater than 1 for these families. Across all traits and families, the AB method exhibited an average improvement of 7.0% over the GBLUP method (RE = 1.070, **Table B5** and **Figure C5**).

Regarding COR metric, the AB method demonstrated superior performance in 7 out of 10 families, as indicated by RE values greater than 1 for these families. However, across all traits and families, the gain of the AB method over the GBLUP method was modest at 1.5% (RE = 1.015).

Additionally, the analysis of NRMSE values revealed that the AB method outperformed the GBLUP method in 5 out of the 10 families, with RE values greater than 1 for these cases. Across all traits and families, the AB method demonstrated a 3.2% improvement in prediction performance, as indicated by an RE value of 1.032 (**Figure C5**, **Table B5**).

In conclusion, the results consistently demonstrate that the AB method outperforms the GBLUP method across various traits, families, and metrics in Data 8 (Soybean_3). These results indicate that method AB is slightly more effective in predicting untested families using tested families as training data.

**Data 10** (Maize_Bi_2018)

In this section, we conducted a comprehensive comparison between the GBLUP and AB methods across different traits for the "Maize_Bi_2018" dataset. The evaluation involved three key metrics: Mean Squared Error (MSE), Correlation (COR), and Normalized Root Mean Squared Error (NRMSE). For each metric, we computed the relative efficiency (RE) to compare the performance of the GBLUP and AB methods (**Figure C6** and **Table B6**).

Regarding the MSE metric, our findings indicate that the AB method outperformed the GBLUP method in 16 out of the 29 families, as evidenced by RE values greater than 1 for these families. Across all traits and families, the AB method exhibited an average improvement of 1.7% over the GBLUP method (RE = 1.017, **Table B6** and **Figure C6**).

Furthermore, regarding the COR metric, the AB method demonstrated superior performance in 14 out of 29 families, as indicated by RE values greater than 1 for these families. However, when considering the metric COR across all traits and families, no significant gain was observed for the AB method over the GBLUP method (RE = 0.992, **Figure C6**, **Table B6**). In other words, the GBLUP method was marginally better than the AB method by 0.8%.

Additionally, the analysis of NRMSE values revealed that the AB method outperformed the GBLUP method in 26 out of the 29 families, with RE values greater than 1 for these cases. Across all traits and families, the AB method displayed a notable 6.2% improvement in prediction performance, as indicated by an RE value of 1.062 (**Figure C6,** **Table B6**).

In summary, the results consistently proved that the AB method outperforms the GBLUP method across various traits, families, and metrics in Data10. Method AB is slightly more effective in predicting untested families using tested families as training data.

| **Table B1**. Data 4. (Maize_3). Prediction accuracy in terms of mean square error (MSE), Pearson´s correlation (COR) and Normalized mean square error (NRMSE). The metrics that end with _C denote the results under the conventional method, while those that end with _AB denote the Adversarial Validation method with variable selection using the Boruta algorithm. RE denotes the relative efficiency for each metric, RE_MSE and RE_NRMSE were computed dividing the MSE_GBLUP by the MSE_AB, and the NRMSE_GBLUP by the NRMSE_AB, while the RE_COR was computed dividing the COR_AB by the COR_GBLUP. RE values larger than one indicate that the AB method outperformed the GBLUP method. | | | | | | | | | | |
| --- | --- | --- | --- | --- | --- | --- | --- | --- | --- | --- |
| Data_set | Family | MSE_GBLUP | COR_GBLUP | NRMSE_GBLUP | MSE_AB | COR_AB | NRMSE_AB | RE_MSE | RE_COR | RE_NRMSE |
| Maize_3 | Z011 | 5728.650 | 0.487 | 1.358 | 6139.745 | 0.509 | 1.310 | 0.933 | 1.046 | 1.037 |
| Maize_3 | Z012 | 4139.803 | 0.508 | 1.101 | 2368.305 | 0.596 | 0.866 | 1.748 | 1.173 | 1.271 |
| Maize_3 | Z013 | 2189.378 | 0.532 | 0.960 | 1716.580 | 0.643 | 0.838 | 1.275 | 1.208 | 1.146 |
| Maize_3 | Z014 | 2117.622 | 0.525 | 1.017 | 1449.272 | 0.633 | 0.844 | 1.461 | 1.207 | 1.205 |
| Maize_3 | Z015 | 1983.694 | 0.551 | 0.935 | 1650.819 | 0.644 | 0.836 | 1.202 | 1.170 | 1.119 |
| Maize_3 | Z016 | 2383.922 | 0.495 | 0.997 | 1619.556 | 0.598 | 0.863 | 1.472 | 1.208 | 1.156 |
| Maize_3 | AF | 3090.511 | 0.516 | 1.061 | 2490.713 | 0.604 | 0.926 | 1.349 | 1.169 | 1.156 |

| **Table B2.** Data 5. (Maize_4). Prediction accuracy in terms of mean square error (MSE), Pearson´s correlation (COR) and Normalized mean square error (NRMSE). The metrics that end with _C denote the results under the conventional method, while those that end with _AB denote the Adversarial Validation method with variable selection using the Boruta algorithm. RE denotes the relative efficiency for each metric, RE_MSE and RE_NRMSE were computed dividing the MSE_GBLUP by the MSE_AB, and the NRMSE_GBLUP by the NRMSE_AB, while the RE_COR was computed dividing the COR_AB by the COR_GBLUP. RE values larger than one indicate that the AB method outperformed the GBLUP method. | | | | | | | | | | |
| --- | --- | --- | --- | --- | --- | --- | --- | --- | --- | --- |
| Data_set | Family | MSE_GBLUP | COR_GBLUP | NRMSE_GBLUP | MSE_AB | COR_AB | NRMSE_AB | RE_MSE | RE_COR | RE_NRMSE |
| Maize_4 | Z016 | 2299.157 | 0.566 | 1.011 | 1699.098 | 0.612 | 0.881 | 1.353 | 1.081 | 1.148 |
| Maize_4 | Z018 | 4481.528 | 0.467 | 1.237 | 3696.363 | 0.590 | 1.165 | 1.212 | 1.265 | 1.061 |
| Maize_4 | Z019 | 5537.821 | 0.600 | 1.270 | 4513.545 | 0.671 | 1.144 | 1.227 | 1.119 | 1.111 |
| Maize_4 | Z020 | 3985.913 | 0.422 | 1.227 | 1882.071 | 0.569 | 0.950 | 2.118 | 1.347 | 1.292 |
| Maize_4 | Z021 | 2156.079 | 0.514 | 1.032 | 1345.889 | 0.640 | 0.886 | 1.602 | 1.245 | 1.164 |
| Maize_4 | Z022 | 2854.216 | 0.506 | 1.006 | 1775.308 | 0.618 | 0.817 | 1.608 | 1.220 | 1.230 |
| Maize_4 | AF | 3552.452 | 0.512 | 1.130 | 2485.379 | 0.617 | 0.974 | 1.520 | 1.213 | 1.168 |

| **Table B3.** Data 6 (Soybean_1). Prediction accuracy in terms of mean square error (MSE), Pearson´s correlation (COR) and Normalized mean square error (NRMSE). The metrics that end with _C denote the results under the conventional method, while those that end with _AB denote the Adversarial Validation method with variable selection using the Boruta algorithm. RE denotes the relative efficiency for each metric, RE_MSE and RE_NRMSE were computed dividing the MSE_GBLUP by the MSE_AB, and the NRMSE_GBLUP by the NRMSE_AB, while the RE_COR was computed dividing the COR_AB by the COR_GBLUP. RE values larger than one indicate that the AB method outperformed the GBLUP method. | | | | | | | | | | |
| --- | --- | --- | --- | --- | --- | --- | --- | --- | --- | --- |
| Data_set | Family | MSE_GBLUP | COR_GBLUP | NRMSE_GBLUP | MSE_AB | COR_AB | NRMSE_AB | RE_MSE | RE_COR | RE_NRMSE |
| Soybean_1 | 10 | 69506.786 | 0.806 | 0.534 | 69819.067 | 0.821 | 0.571 | 0.996 | 1.020 | 0.936 |
| Soybean_1 | 11 | 75028.774 | 0.755 | 0.611 | 72109.313 | 0.813 | 0.556 | 1.040 | 1.078 | 1.100 |
| Soybean_1 | 12 | 71630.671 | 0.776 | 0.677 | 67120.832 | 0.823 | 0.697 | 1.067 | 1.060 | 0.972 |
| Soybean_1 | 2 | 68306.216 | 0.808 | 0.600 | 65703.599 | 0.853 | 0.504 | 1.040 | 1.056 | 1.189 |
| Soybean_1 | 3 | 73597.906 | 0.816 | 0.541 | 72772.257 | 0.852 | 0.485 | 1.011 | 1.044 | 1.115 |
| Soybean_1 | 4 | 78141.017 | 0.801 | 0.548 | 75258.616 | 0.844 | 0.502 | 1.038 | 1.054 | 1.091 |
| Soybean_1 | 5 | 73591.859 | 0.819 | 0.575 | 68513.699 | 0.851 | 0.507 | 1.074 | 1.038 | 1.135 |
| Soybean_1 | 6 | 63812.652 | 0.826 | 0.527 | 64710.193 | 0.842 | 0.529 | 0.986 | 1.020 | 0.997 |
| Soybean_1 | 8 | 69240.305 | 0.813 | 0.624 | 68013.282 | 0.847 | 0.503 | 1.018 | 1.042 | 1.240 |
| Soybean_1 | 9 | 77737.162 | 0.786 | 0.586 | 71982.597 | 0.827 | 0.555 | 1.080 | 1.052 | 1.056 |
| Soybean_1 | AF | 72059.335 | 0.801 | 0.582 | 69600.346 | 0.837 | 0.541 | 1.035 | 1.046 | 1.083 |

| **Table B4**. Data 7 (Soybean_2)**.** Prediction accuracy in terms of mean square error (MSE), Pearson´s correlation (COR) and Normalized mean square error (NRMSE). The metrics that end with _C denote the results under the conventional method, while those that end with _AB denote the Adversarial Validation method with variable selection using the Boruta algorithm. RE denotes the relative efficiency for each metric, RE_MSE and RE_NRMSE were computed dividing the MSE_GBLUP by the MSE_AB, and the NRMSE_GBLUP by the NRMSE_AB, while the RE_COR was computed dividing the COR_AB by the COR_GBLUP. RE values larger than one indicate that the AB method outperformed the GBLUP method. | | | | | | | | | | |
| --- | --- | --- | --- | --- | --- | --- | --- | --- | --- | --- |
| Data_set | Family | MSE_GBLUP | COR_GBLUP | NRMSE_GBLUP | MSE_AB | COR_AB | NRMSE_AB | RE_MSE | RE_COR | RE_NRMSE |
| Soybean_2 | 13 | 54764.280 | 0.703 | 0.818 | 50818.120 | 0.789 | 0.647 | 1.078 | 1.122 | 1.264 |
| Soybean_2 | 14 | 56877.896 | 0.701 | 0.784 | 57707.361 | 0.767 | 0.768 | 0.986 | 1.094 | 1.021 |
| Soybean_2 | 15 | 55232.424 | 0.688 | 0.739 | 59894.058 | 0.760 | 0.654 | 0.922 | 1.106 | 1.129 |
| Soybean_2 | 17 | 63696.893 | 0.683 | 0.829 | 61364.646 | 0.776 | 0.727 | 1.038 | 1.136 | 1.141 |
| Soybean_2 | 22 | 61933.744 | 0.643 | 0.816 | 63061.555 | 0.744 | 0.673 | 0.982 | 1.157 | 1.211 |
| Soybean_2 | 23 | 74069.416 | 0.689 | 0.791 | 75639.031 | 0.783 | 0.959 | 0.979 | 1.138 | 0.824 |
| Soybean_2 | 24 | 70870.393 | 0.700 | 0.741 | 63648.513 | 0.765 | 0.638 | 1.113 | 1.093 | 1.161 |
| Soybean_2 | 25 | 93088.246 | 0.658 | 0.759 | 90870.017 | 0.717 | 0.664 | 1.024 | 1.090 | 1.142 |
| Soybean_2 | 26 | 72155.960 | 0.884 | 0.786 | 51313.980 | 0.884 | 0.500 | 1.406 | 1.000 | 1.571 |
| Soybean_2 | 27 | 49808.819 | 0.795 | 0.621 | 52679.362 | 0.860 | 0.543 | 0.946 | 1.082 | 1.143 |
| Soybean_2 | AF | 65249.807 | 0.714 | 0.768 | 62699.664 | 0.785 | 0.677 | 1.047 | 1.102 | 1.161 |

| **Table B5.** Data 8 (Soybean_3)**.** Prediction accuracy in terms of mean square error (MSE), Pearson´s correlation (COR) and Normalized mean square error (NRMSE). The metrics that end with _C denote the results under the conventional method, while those that end with _AB denote the Adversarial Validation method with variable selection using the Boruta algorithm. RE denotes the relative efficiency for each metric, RE_MSE and RE_NRMSE were computed dividing the MSE_GBLUP by the MSE_AB, and the NRMSE_GBLUP by the NRMSE_AB, while the RE_COR was computed dividing the COR_AB by the COR_GBLUP. RE values larger than one indicate that the AB method outperformed the GBLUP method. | | | | | | | | | | |
| --- | --- | --- | --- | --- | --- | --- | --- | --- | --- | --- |
| Data_set | Family | MSE_GBLUP | COR_GBLUP | NRMSE_GBLUP | MSE_AB | COR_AB | NRMSE_AB | RE_MSE | RE_COR | RE_NRMSE |
| Soybean_3 | 28 | 66075.446 | 0.790 | 0.681 | 52577.048 | 0.796 | 0.688 | 1.257 | 1.008 | 0.989 |
| Soybean_3 | 29 | 40531.068 | 0.818 | 0.541 | 47377.246 | 0.884 | 0.469 | 0.855 | 1.081 | 1.153 |
| Soybean_3 | 30 | 75267.622 | 0.837 | 0.495 | 67813.496 | 0.836 | 0.496 | 1.110 | 1.000 | 0.999 |
| Soybean_3 | 31 | 136132.030 | 0.838 | 0.548 | 111052.467 | 0.815 | 0.549 | 1.226 | 0.973 | 0.998 |
| Soybean_3 | 32 | 52156.395 | 0.809 | 0.604 | 57799.124 | 0.857 | 0.497 | 0.902 | 1.059 | 1.215 |
| Soybean_3 | 33 | 110989.887 | 0.794 | 0.581 | 123487.156 | 0.723 | 0.641 | 0.899 | 0.911 | 0.907 |
| Soybean_3 | 34 | 107390.476 | 0.831 | 0.514 | 71010.171 | 0.876 | 0.496 | 1.512 | 1.054 | 1.036 |
| Soybean_3 | 36 | 152783.663 | 0.705 | 0.671 | 147805.566 | 0.739 | 0.648 | 1.034 | 1.048 | 1.036 |
| Soybean_3 | 37 | 87420.013 | 0.752 | 0.669 | 92886.426 | 0.791 | 0.629 | 0.941 | 1.053 | 1.064 |
| Soybean_3 | 38 | 77413.540 | 0.793 | 0.566 | 80061.216 | 0.764 | 0.612 | 0.967 | 0.964 | 0.926 |
| Soybean_3 | AF | 90616.014 | 0.796 | 0.587 | 85186.992 | 0.808 | 0.572 | 1.070 | 1.015 | 1.032 |

| **Table B6.** Data 10 (Maize_Bi_2018)**.** Prediction accuracy in terms of mean square error (MSE), Pearson´s correlation (COR) and Normalized mean square error (NRMSE). The metrics that end with _C denote the results under the conventional method, while those that end with _AB denote the Adversarial Validation method with variable selection using the Boruta algorithm. RE denotes the relative efficiency for each metric, RE_MSE and RE_NRMSE were computed dividing the MSE_GBLUP by the MSE_AB, and the NRMSE_GBLUP by the NRMSE_AB, while the RE_COR was computed dividing the COR_AB by the COR_GBLUP. RE values larger than one indicate that the AB method outperformed the GBLUP method. | | | | | | | | | | |
| --- | --- | --- | --- | --- | --- | --- | --- | --- | --- | --- |
| Data_set | Family | MSE_GBLUP | COR_GBLUP | NRMSE_GBLUP | MSE_AB | COR_AB | NRMSE_AB | RE_MSE | RE_COR | RE_NRMSE |
| Maize_BI_CIMMYT | 1 | 23.707 | 0.131 | 1.388 | 23.616 | 0.110 | 1.334 | 1.004 | 0.842 | 1.040 |
| Maize_BI_CIMMYT | 10 | 23.557 | 0.306 | 1.167 | 21.600 | 0.299 | 1.145 | 1.091 | 0.978 | 1.019 |
| Maize_BI_CIMMYT | 11 | 41.159 | 0.258 | 1.339 | 41.995 | 0.236 | 1.354 | 0.980 | 0.915 | 0.989 |
| Maize_BI_CIMMYT | 13 | 23.059 | 0.114 | 1.683 | 23.047 | 0.119 | 1.509 | 1.001 | 1.047 | 1.116 |
| Maize_BI_CIMMYT | 14 | 20.930 | 0.327 | 1.298 | 22.874 | 0.334 | 1.298 | 0.915 | 1.022 | 1.000 |
| Maize_BI_CIMMYT | 15 | 69.915 | 0.218 | 2.096 | 62.180 | 0.226 | 1.842 | 1.124 | 1.039 | 1.138 |
| Maize_BI_CIMMYT | 16 | 33.612 | 0.188 | 1.416 | 29.534 | 0.177 | 1.342 | 1.138 | 0.941 | 1.055 |
| Maize_BI_CIMMYT | 17 | 25.216 | 0.222 | 1.369 | 22.698 | 0.219 | 1.276 | 1.111 | 0.985 | 1.072 |
| Maize_BI_CIMMYT | 18 | 56.356 | 0.176 | 1.386 | 47.566 | 0.189 | 1.335 | 1.185 | 1.074 | 1.038 |
| Maize_BI_CIMMYT | 19 | 66.060 | 0.148 | 1.343 | 60.613 | 0.156 | 1.332 | 1.090 | 1.048 | 1.008 |
| Maize_BI_CIMMYT | 2 | 72.889 | 0.187 | 1.292 | 75.699 | 0.195 | 1.301 | 0.963 | 1.043 | 0.993 |
| Maize_BI_CIMMYT | 20 | 44.322 | 0.181 | 1.459 | 41.083 | 0.175 | 1.389 | 1.079 | 0.971 | 1.050 |
| Maize_BI_CIMMYT | 27 | 20.513 | 0.287 | 1.360 | 19.949 | 0.282 | 1.350 | 1.028 | 0.981 | 1.007 |
| Maize_BI_CIMMYT | 28 | 73.045 | 0.134 | 6.186 | 65.638 | 0.148 | 6.056 | 1.113 | 1.102 | 1.021 |
| Maize_BI_CIMMYT | 29 | 53.339 | 0.071 | 1.770 | 53.434 | 0.053 | 1.762 | 0.998 | 0.740 | 1.005 |
| Maize_BI_CIMMYT | 3 | 20.656 | 0.187 | 1.274 | 21.257 | 0.198 | 1.245 | 0.972 | 1.057 | 1.024 |
| Maize_BI_CIMMYT | 30 | 20.611 | 0.210 | 1.303 | 21.140 | 0.205 | 1.253 | 0.975 | 0.975 | 1.039 |
| Maize_BI_CIMMYT | 31 | 16.455 | 0.212 | 1.425 | 16.342 | 0.212 | 1.380 | 1.007 | 1.001 | 1.033 |
| Maize_BI_CIMMYT | 32 | 28.522 | 0.283 | 1.299 | 28.009 | 0.284 | 1.269 | 1.018 | 1.002 | 1.024 |
| Maize_BI_CIMMYT | 33 | 21.100 | 0.113 | 66.044 | 24.136 | 0.107 | 39.667 | 0.874 | 0.948 | 1.665 |
| Maize_BI_CIMMYT | 38 | 38.566 | 0.202 | 1.602 | 42.276 | 0.213 | 1.559 | 0.912 | 1.053 | 1.028 |
| Maize_BI_CIMMYT | 39 | 18.905 | 0.077 | 1.527 | 19.143 | 0.085 | 1.508 | 0.988 | 1.096 | 1.013 |
| Maize_BI_CIMMYT | 4 | 21.280 | 0.255 | 1.199 | 21.090 | 0.249 | 1.182 | 1.009 | 0.976 | 1.015 |
| Maize_BI_CIMMYT | 40 | 58.495 | 0.064 | 1.772 | 54.639 | 0.068 | 1.508 | 1.071 | 1.060 | 1.175 |
| Maize_BI_CIMMYT | 5 | 22.253 | 0.265 | 1.169 | 25.030 | 0.251 | 1.173 | 0.889 | 0.946 | 0.997 |
| Maize_BI_CIMMYT | 6 | 23.583 | 0.326 | 1.422 | 24.744 | 0.317 | 1.345 | 0.953 | 0.972 | 1.057 |
| Maize_BI_CIMMYT | 7 | 17.372 | 0.354 | 1.280 | 17.688 | 0.331 | 1.266 | 0.982 | 0.936 | 1.012 |
| Maize_BI_CIMMYT | 8 | 30.109 | 0.269 | 1.219 | 30.736 | 0.273 | 1.215 | 0.980 | 1.016 | 1.003 |
| Maize_BI_CIMMYT | 9 | 34.489 | 0.149 | 3.029 | 33.199 | 0.148 | 2.588 | 1.039 | 0.989 | 1.171 |
| Maize_BI_CIMMYT | AF | 35.175 | 0.204 | 3.866 | 34.171 | 0.202 | 2.889 | 1.017 | 0.992 | 1.062 |

| **Table B7.** Data 11 (Maize_Bi_2019_D)**.** Prediction accuracy in terms of mean square error (MSE), Pearson´s correlation (COR) and Normalized mean square error (NRMSE). The metrics that end with _C denote the results under the conventional method, while those that end with _AB denote the Adversarial Validation method with variable selection using the Boruta algorithm. RE denotes the relative efficiency for each metric, RE_MSE and RE_NRMSE were computed dividing the MSE_GBLUP by the MSE_AB, and the NRMSE_GBLUP by the NRMSE_AB, while the RE_COR was computed dividing the COR_AB by the COR_GBLUP. RE values larger than one indicate that the AB method outperformed the GBLUP method. | | | | | | | | | | |
| --- | --- | --- | --- | --- | --- | --- | --- | --- | --- | --- |
| Data_set | Family | MSE_GBLUP | COR_GBLUP | NRMSE_GBLUP | MSE_AB | COR_AB | NRMSE_AB | RE_MSE | RE_COR | RE_NRMSE |
|  |  |  |  |  |  |  |  |  |  |  |
| Maize_BI_2019_D | F1 | 20.287 | 0.137 | 1.113 | 20.195 | 0.137 | 1.084 | 1.005 | 0.999 | 1.025 |
| Maize_BI_2019_D | F2 | 28.563 | 0.102 | 5.756 | 28.650 | 0.093 | 6.686 | 0.997 | 0.906 | 1.009 |
| Maize_BI_2019_D | F3 | 33.939 | 0.095 | 1.120 | 33.594 | 0.091 | 1.107 | 1.010 | 0.952 | 1.010 |
| Maize_BI_2019_D | F4 | 40.570 | 0.113 | 1.328 | 42.321 | 0.092 | 1.331 | 0.959 | 0.821 | 0.998 |
| Maize_BI_2019_D | F5 | 23.760 | 0.081 | 4.550 | 24.246 | 0.080 | 3.965 | 0.980 | 0.992 | 1.024 |
| Maize_BI_2019_D | F6 | 18.573 | 0.210 | 6.196 | 17.465 | 0.197 | 6.176 | 1.063 | 0.938 | 0.994 |
| Maize_BI_2019_D | F7 | 27.082 | 0.169 | 1.610 | 27.348 | 0.154 | 1.669 | 0.990 | 0.914 | 0.992 |
| Maize_BI_2019_D | F8 | 15.531 | 0.154 | 20.451 | 14.984 | 0.165 | 17.803 | 1.037 | 1.076 | 1.017 |
| Maize_BI_2019_D | F9 | 13.718 | 0.146 | 1.088 | 12.861 | 0.145 | 1.059 | 1.067 | 0.993 | 1.027 |
| Maize_BI_2019_D | F10 | 27.939 | 0.035 | 1.235 | 26.000 | 0.027 | 1.206 | 1.075 | 0.775 | 1.021 |
| Maize_BI_2019_D | F11 | 11.041 | 0.282 | 1.013 | 11.174 | 0.272 | 1.015 | 0.988 | 0.964 | 0.997 |
| Maize_BI_2019_D | F12 | 19.382 | 0.167 | 0.968 | 18.928 | 0.163 | 0.967 | 1.024 | 0.973 | NA |
| Maize_BI_2019_D | F13 | 19.949 | 0.123 | 1.188 | 20.422 | 0.132 | 1.188 | 0.977 | 1.075 | 0.999 |
| Maize_BI_2019_D | F14 | 63.639 | 0.160 | 27.141 | 66.030 | 0.163 | 22.931 | 0.964 | 1.018 | 1.030 |
| Maize_BI_2019_D | F15 | 23.963 | 0.156 | 1.190 | 21.779 | 0.153 | 1.163 | 1.100 | 0.982 | 1.022 |
| Maize_BI_2019_D | F16 | 33.091 | 0.094 | 1.162 | 32.896 | 0.100 | 1.148 | 1.006 | 1.075 | 1.011 |
| Maize_BI_2019_D | F17 | 57.241 | 0.200 | 757.503 | 56.026 | 0.200 | 698.247 | 1.022 | 1.000 | 1.006 |
| Maize_BI_2019_D | F18 | 33.016 | -0.157 | 4.031 | 33.842 | -0.142 | 3.700 | 0.976 | 0.903 | 0.997 |
| Maize_BI_2019_D | F19 | 17.344 | 0.071 | 7.489 | 17.594 | 0.065 | 6.089 | 0.986 | 0.910 | 1.015 |
| Maize_BI_2019_D | AF | 27.823 | 0.123 | 44.533 | 27.703 | 0.120 | 40.975 | 1.012 | 0.961 | 1.011 |

| **Table B8.** Data 12 (Maize_Bi_2019_O). Prediction accuracy in terms of mean square error (MSE), Pearson´s correlation (COR) and Normalized mean square error (NRMSE). The metrics that end with _C denote the results under the conventional method, while those that end with _AB denote the Adversarial Validation method with variable selection using the Boruta algorithm. RE denotes the relative efficiency for each metric, RE_MSE and RE_NRMSE were computed dividing the MSE_GBLUP by the MSE_AB, and the NRMSE_GBLUP by the NRMSE_AB, while the RE_COR was computed dividing the COR_AB by the COR_GBLUP. RE values larger than one indicate that the AB method outperformed the GBLUP method. | | | | | | | | | | |
| --- | --- | --- | --- | --- | --- | --- | --- | --- | --- | --- |
| Data_set | Family | MSE_GBLUP | COR_GBLUP | NRMSE_GBLUP | MSE_AB | COR_AB | NRMSE_AB | RE_MSE | RE_COR | RE_NRMSE |
| Maize_BI_2019_O | F1 | 10.593 | 0.169 | 1.051 | 10.784 | 0.145 | 1.061 | 0.982 | 0.858 | 0.991 |
| Maize_BI_2019_O | F2 | 18.385 | 0.197 | 1.131 | 19.163 | 0.175 | 1.140 | 0.959 | 0.886 | 0.992 |
| Maize_BI_2019_O | F3 | 12.646 | 0.155 | 1.130 | 12.623 | 0.142 | 1.122 | 1.002 | 0.916 | 1.007 |
| Maize_BI_2019_O | F4 | 18.314 | 0.114 | 1.462 | 17.898 | 0.104 | 1.443 | 1.023 | 0.913 | 1.013 |
| Maize_BI_2019_O | F5 | 25.221 | 0.098 | 1.313 | 26.381 | 0.071 | 1.318 | 0.956 | 0.729 | 0.996 |
| Maize_BI_2019_O | F6 | 22.923 | 0.134 | 1.222 | 22.648 | 0.149 | 1.198 | 1.012 | 1.108 | 1.020 |
| Maize_BI_2019_O | F7 | 61.827 | 0.188 | 1.108 | 61.728 | 0.184 | 1.103 | 1.002 | 0.979 | 1.005 |
| Maize_BI_2019_O | F8 | 20.547 | 0.113 | 1.149 | 20.908 | 0.102 | 1.144 | 0.983 | 0.905 | 1.004 |
| Maize_BI_2019_O | F9 | 14.519 | 0.111 | 1.253 | 13.468 | 0.090 | 1.231 | 1.078 | 0.809 | 1.018 |
| Maize_BI_2019_O | F10 | 14.062 | 0.252 | 1.187 | 14.120 | 0.235 | 1.156 | 0.996 | 0.934 | 1.027 |
| Maize_BI_2019_O | F11 | 17.502 | 0.203 | 3.342 | 17.411 | 0.206 | 3.005 | 1.005 | 1.013 | 1.112 |
| Maize_BI_2019_O | F12 | 19.243 | 0.093 | 1.182 | 18.548 | 0.096 | 1.164 | 1.038 | 1.031 | 1.015 |
| Maize_BI_2019_O | F13 | 22.196 | 0.091 | 1.321 | 22.875 | 0.093 | 1.298 | 0.970 | 1.020 | 1.018 |
| Maize_BI_2019_O | F14 | 20.213 | 0.201 | 1.877 | 21.520 | 0.197 | 1.780 | 0.939 | 0.977 | 1.054 |
| Maize_BI_2019_O | F15 | 23.876 | 0.178 | 1.143 | 23.933 | 0.184 | 1.140 | 0.998 | 1.038 | 1.003 |
| Maize_BI_2019_O | F16 | 15.625 | 0.116 | 1.201 | 15.368 | 0.123 | 1.178 | 1.017 | 1.059 | 1.020 |
| Maize_BI_2019_O | F17 | 41.219 | 0.300 | 22.591 | 40.914 | 0.300 | 22.534 | 1.007 | 1.000 | 1.002 |
| Maize_BI_2019_O | F18 | 20.375 | 0.001 | 1.241 | 19.803 | 0.001 | 1.221 | 1.029 | 1.772 | 1.017 |
| Maize_BI_2019_O | F19 | 11.175 | 0.093 | 1.452 | 11.289 | 0.079 | 1.443 | 0.990 | 0.852 | 1.006 |
| Maize_BI_2019_O | AF | 21.603 | 0.148 | 2.492 | 21.652 | 0.140 | 2.457 | 0.999 | 0.592 | 1.017 |

| **A** |
| --- |
| 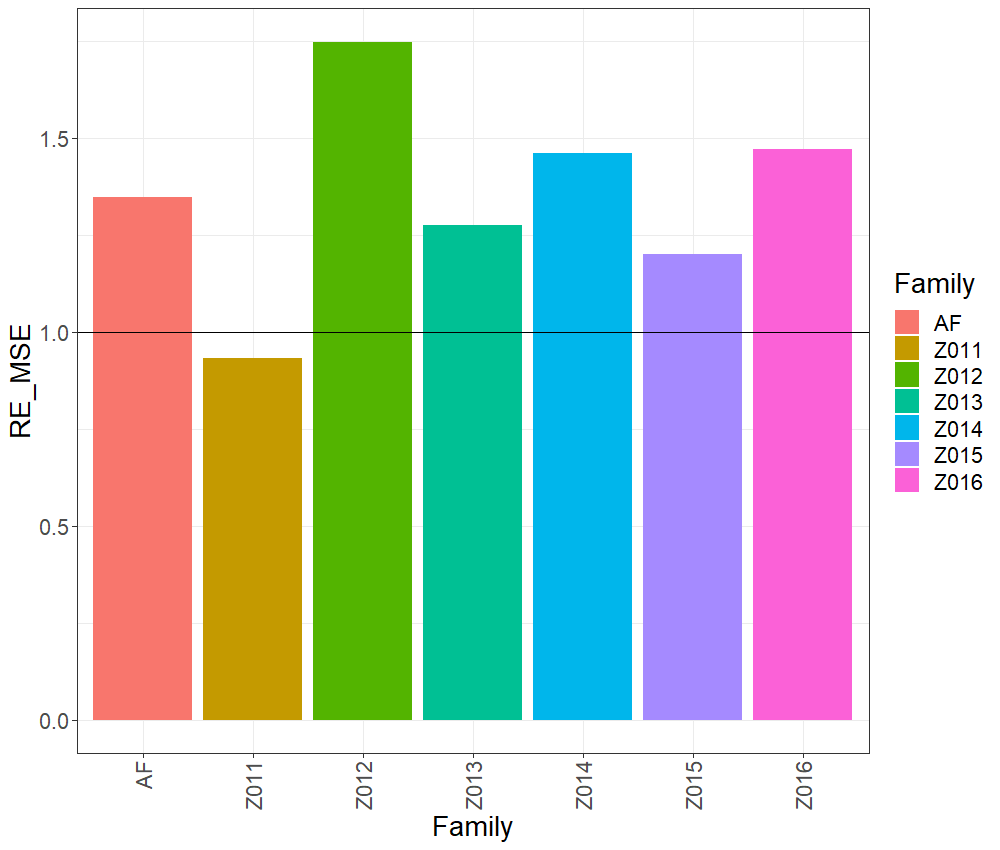 |
| **B** |
| 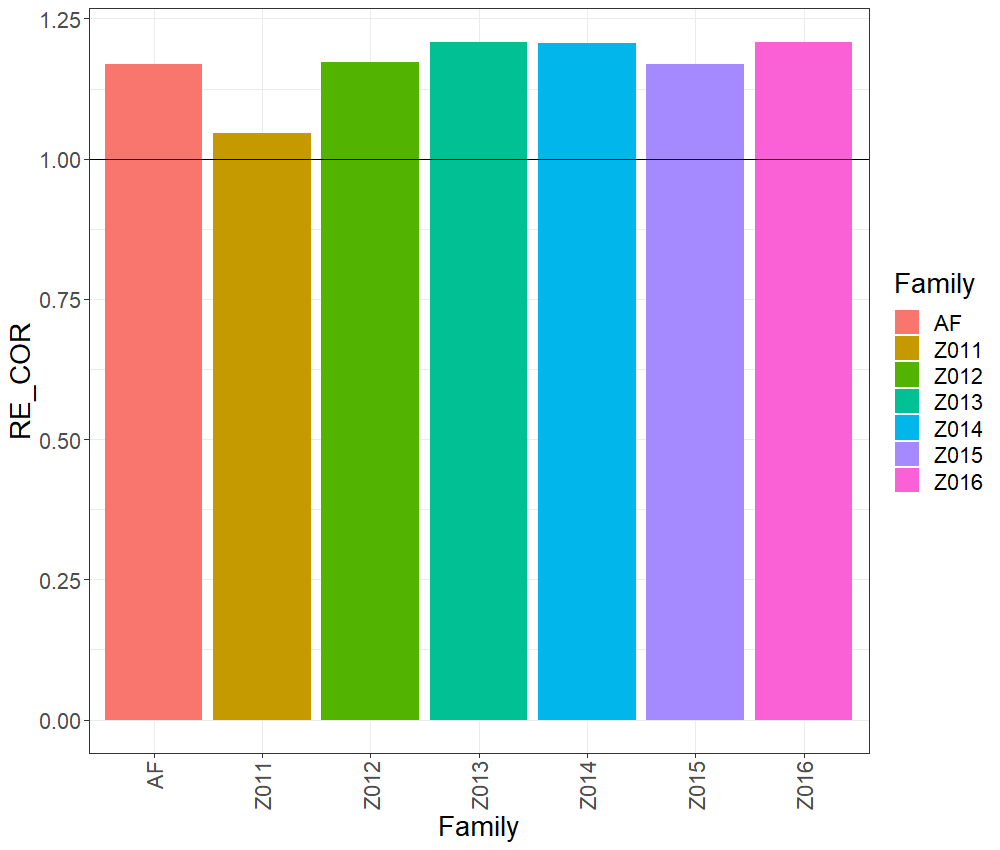 |
| **C** |
| 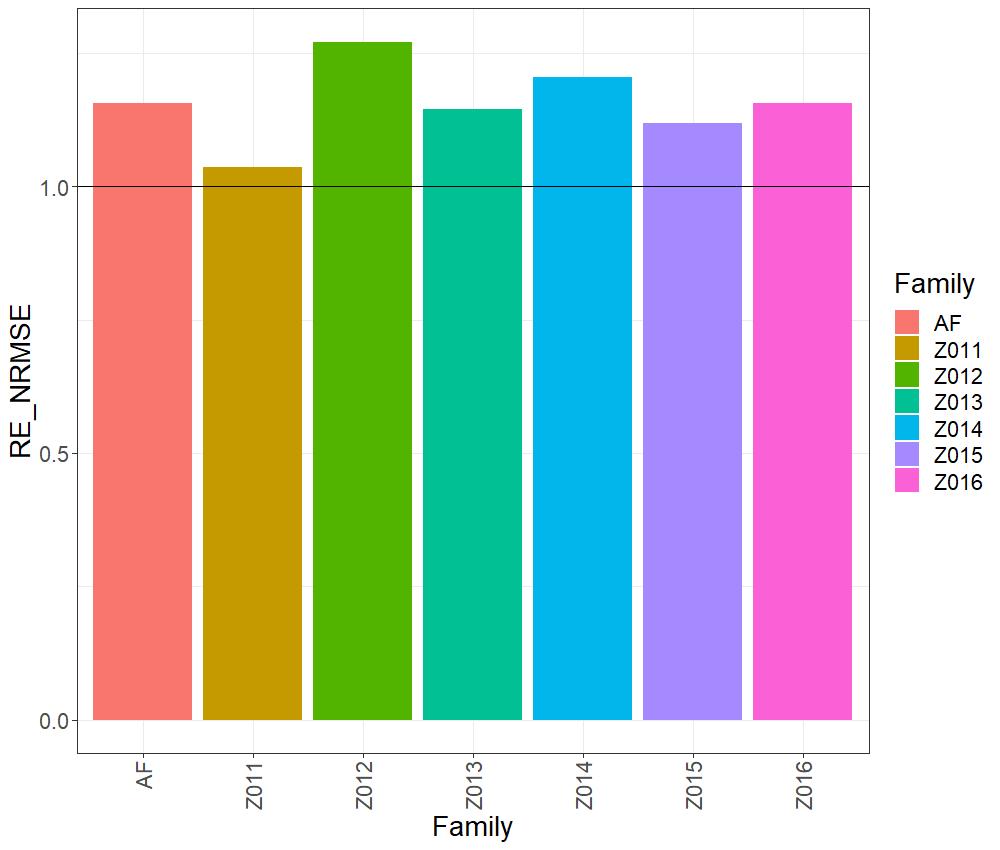 |

**Figure C1.** Data 4 (Maize_3). Relative efficiency (RE) between the proposed method (AB, Adversarial validation with Boruta for variable selection) and the conventional, C, method for each family and across family (AF) in terms of A) Mean square error (MSE), B) Average Pearson´s Correlation (COR) and C) Normalized root mean square error (NRMSE). RE>1 means that the AB method outperformed the GBLUP method, RE<1 means that the GBLUP method outperformed the AB method and RE=1 means that both methods performed equally.

| **A** |
| --- |
| 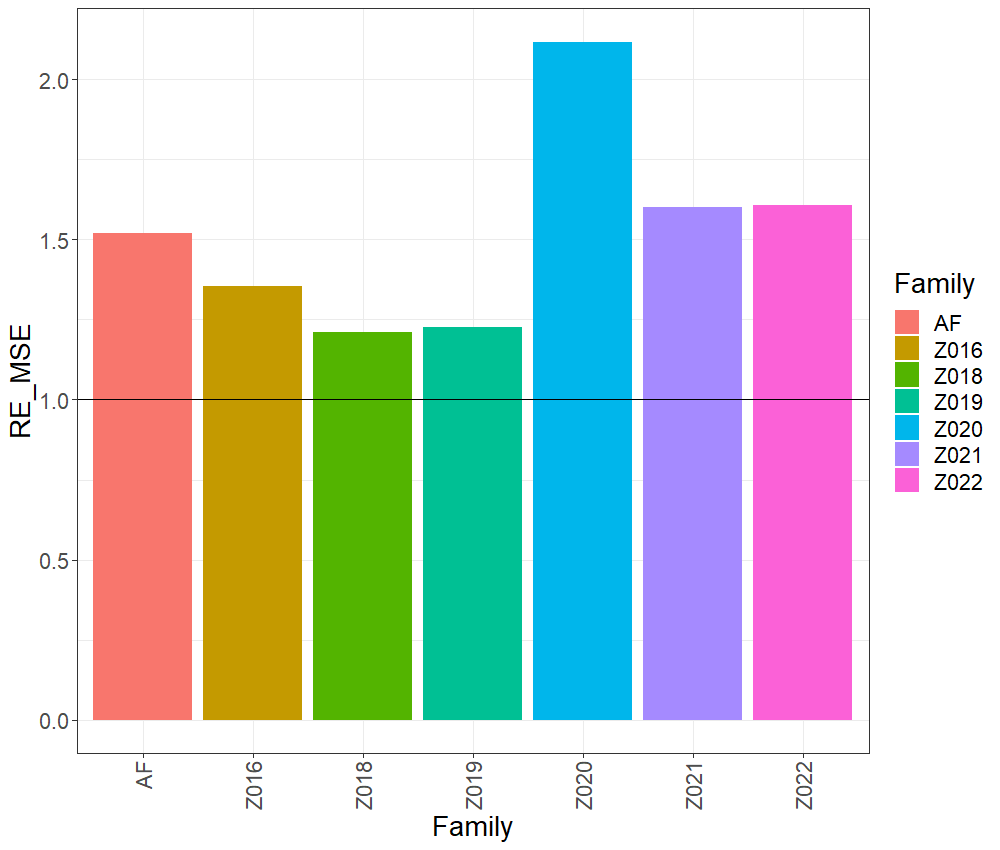 |
| **B** |
| 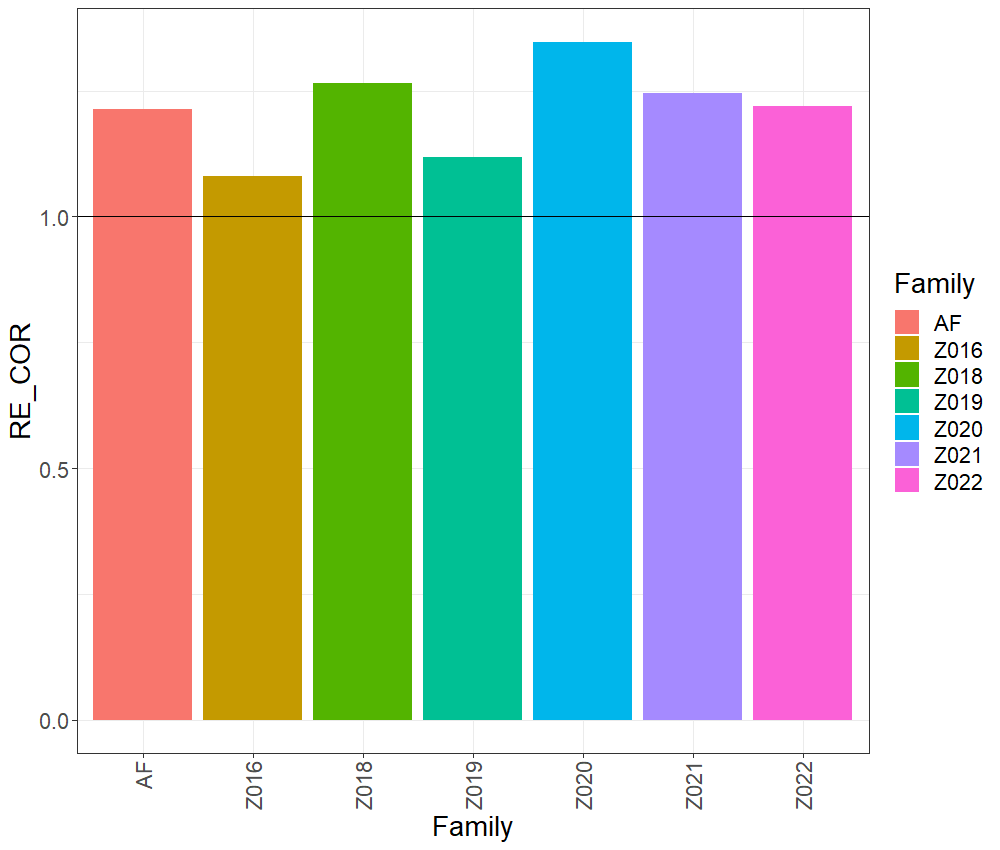 |
| **C** |
| 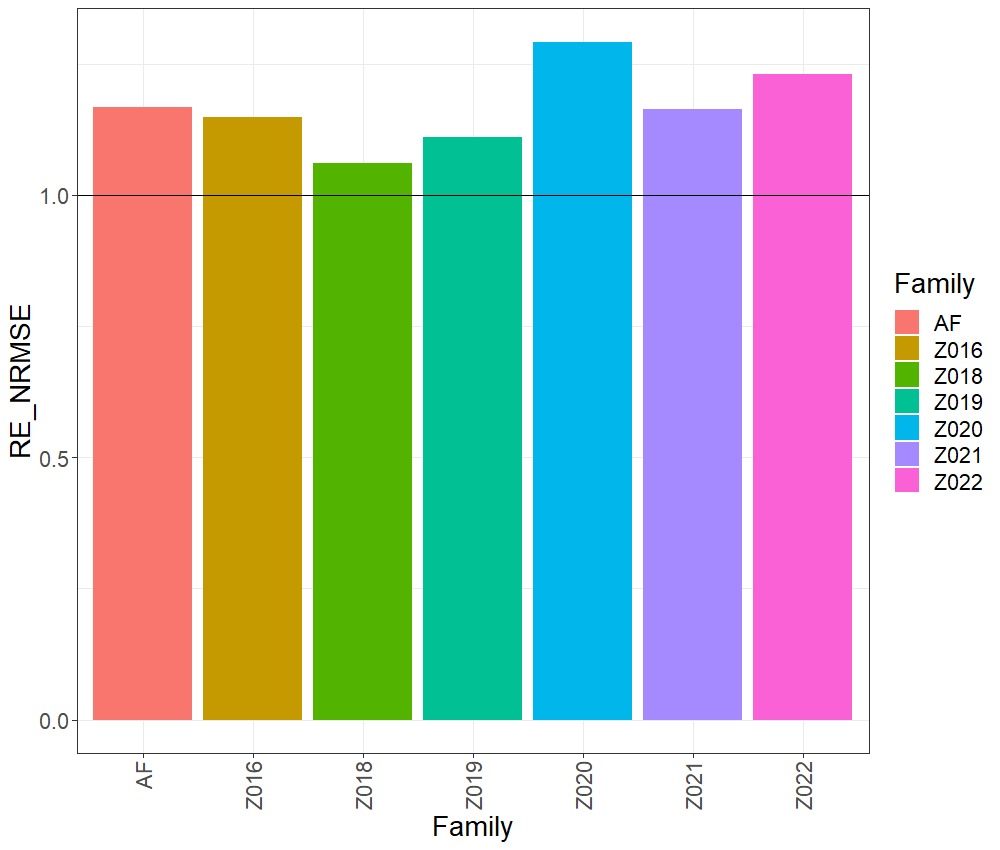 |

**Figure C2**. Data 5 (Maize_4). Relative efficiency (RE) between the proposed method (AB, Adversarial validation with Boruta for variable selection) and the conventional, C, method for each family and across family (AF) in terms of A) Mean square error (MSE), B) Average Pearson´s Correlation (COR) and C) Normalized root mean square error (NRMSE). RE>1 means that the AB method outperformed the GBLUP method, RE<1 means that the GBLUP method outperformed the AB method and RE=1 means that both methods performed equally.

| **A** |
| --- |
| 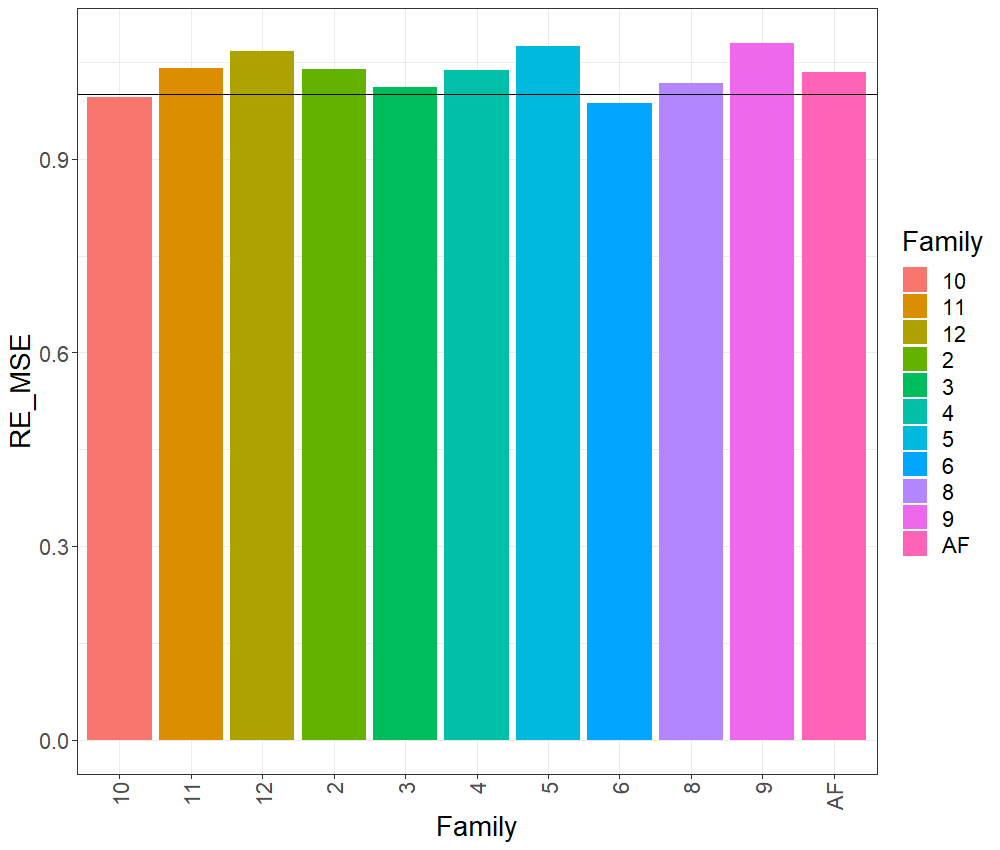 |
| **B** |
| 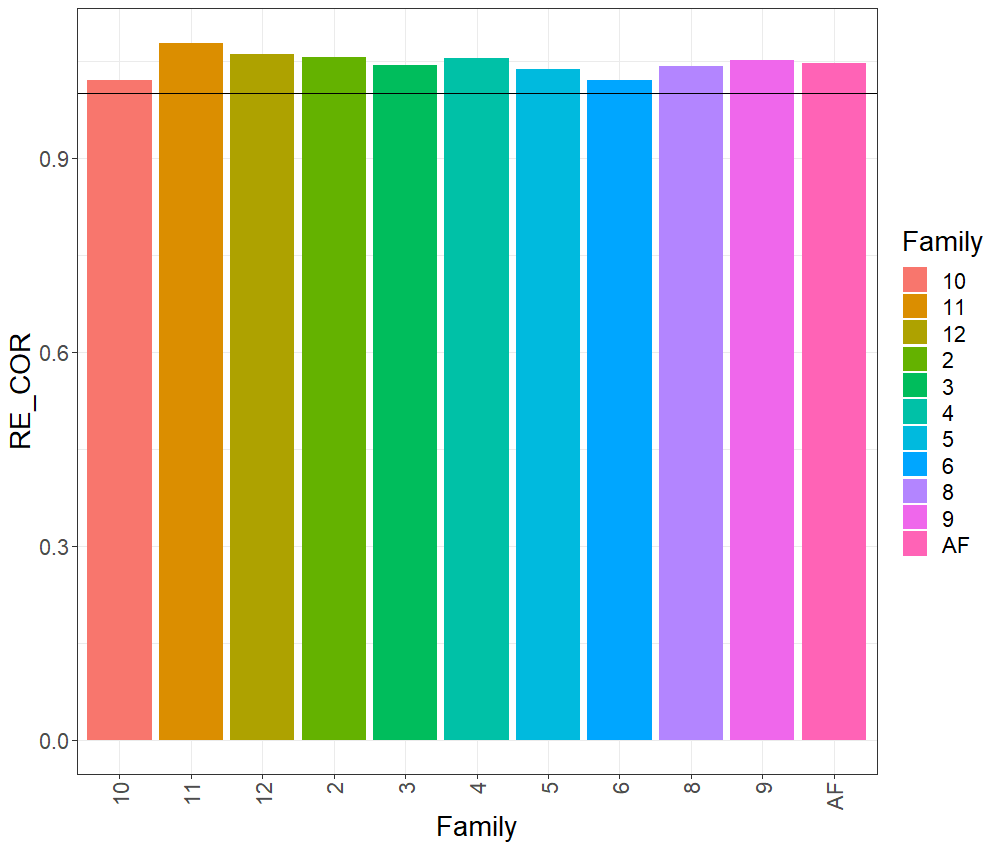 |
| **C** |
| 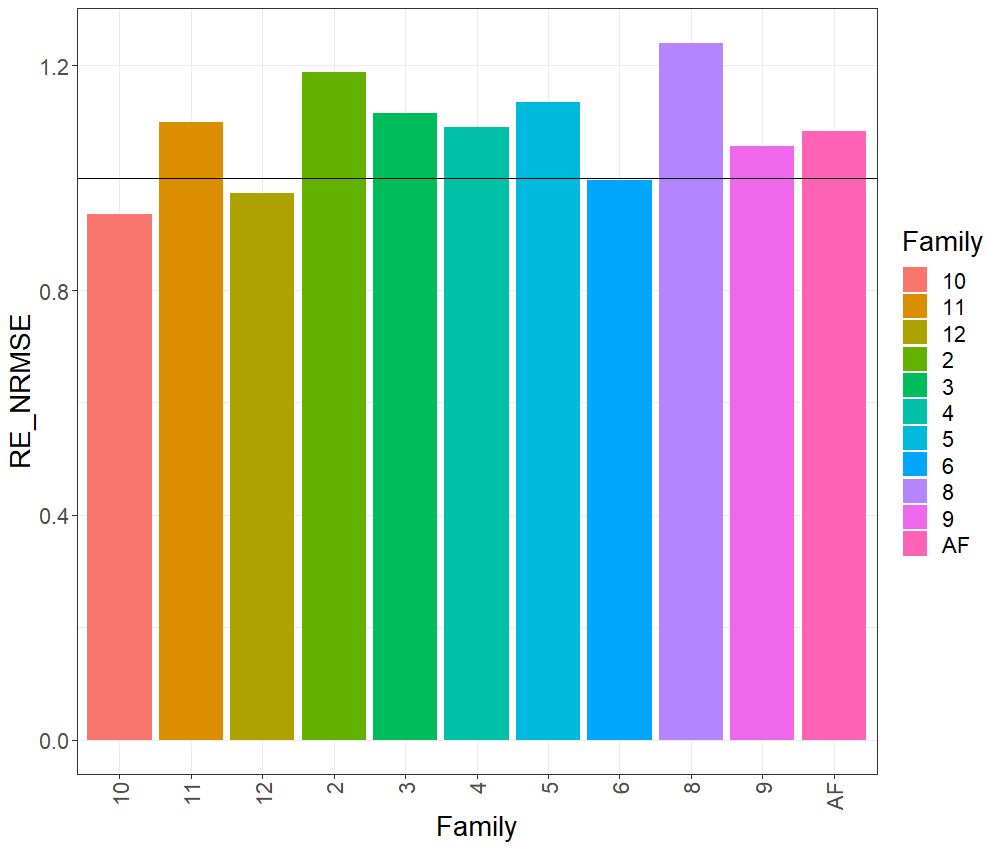 |

**Figure C3**. Data 6 (Soybean_1)**.** Relative efficiency (RE) between the proposed method (AB, Adversarial validation with Boruta for variable selection) and the conventional, C, method for each family and across family (AF) in terms of A) Mean square error (MSE), B) Average Pearson´s Correlation (COR) and C) Normalized root mean square error (NRMSE). RE>1 means that the AB method outperformed the GBLUP method, RE<1 means that the GBLUP method outperformed the AB method and RE=1 means that both methods performed equally.

| **A** |
| --- |
| 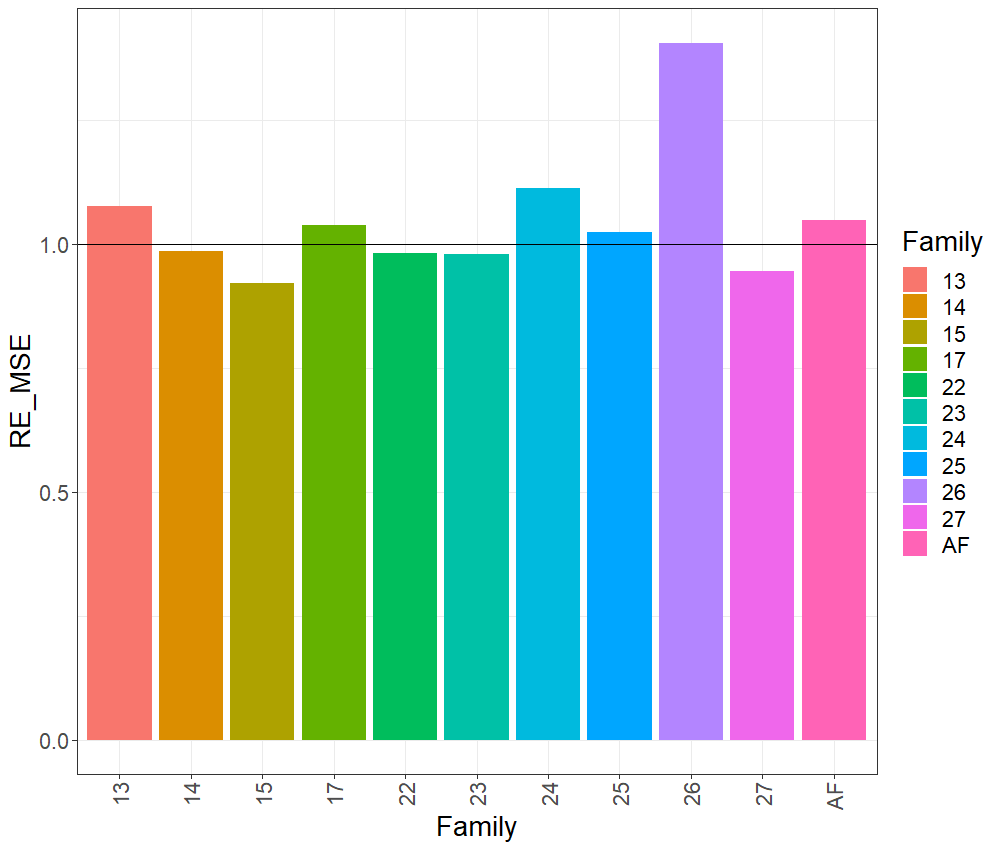 |
| **B** |
| 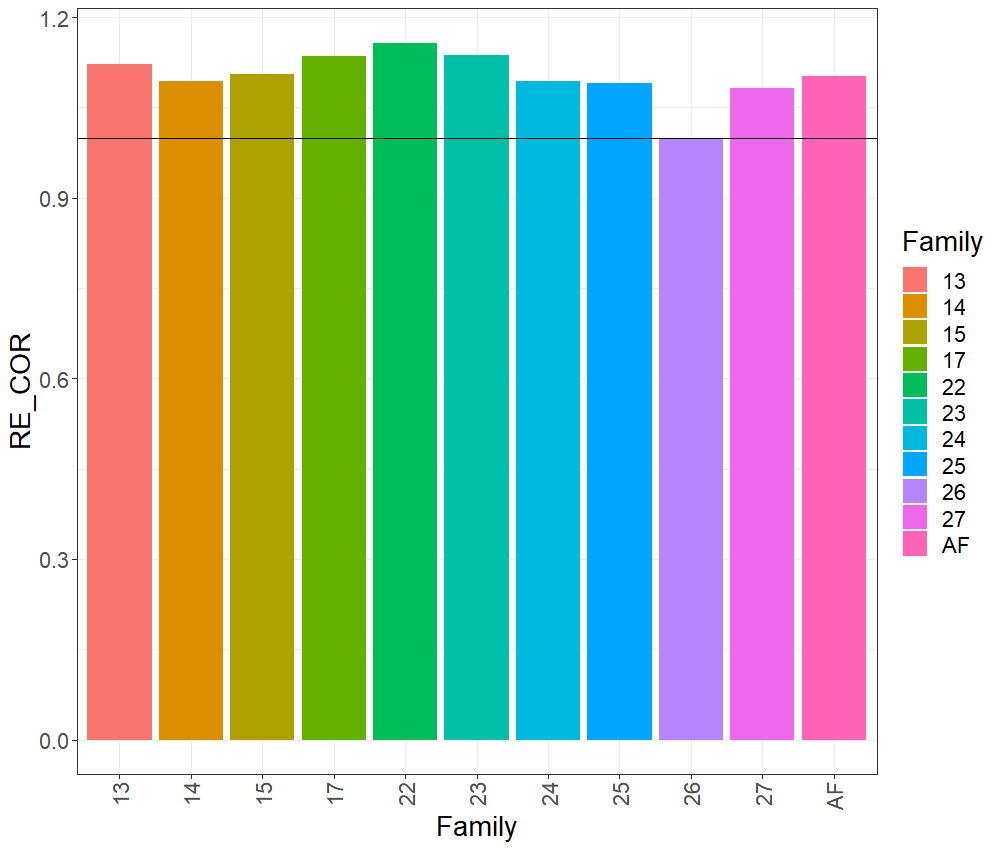 |
| **C** |
| 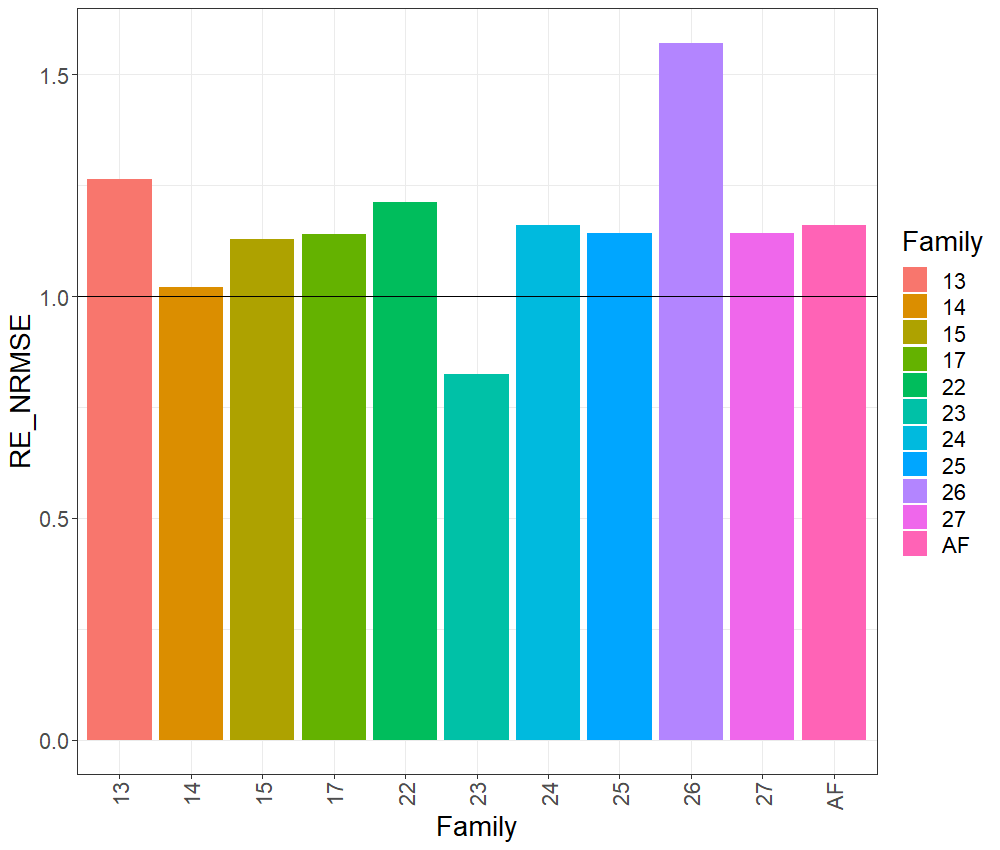 |

**Figure C4**. Data 7 (Soybean_2). Relative efficiency (RE) between the proposed method (AB, Adversarial validation with Boruta for variable selection) and the conventional, C, method for each family and across family (AF) in terms of A) Mean square error (MSE), B) Average Pearson´s Correlation (COR) and C) Normalized root mean square error (NRMSE). RE>1 means that the AB method outperformed the GBLUP method, RE<1 means that the GBLUP method outperformed the AB method and RE=1 means that both methods performed equally.

| **A** |
| --- |
| 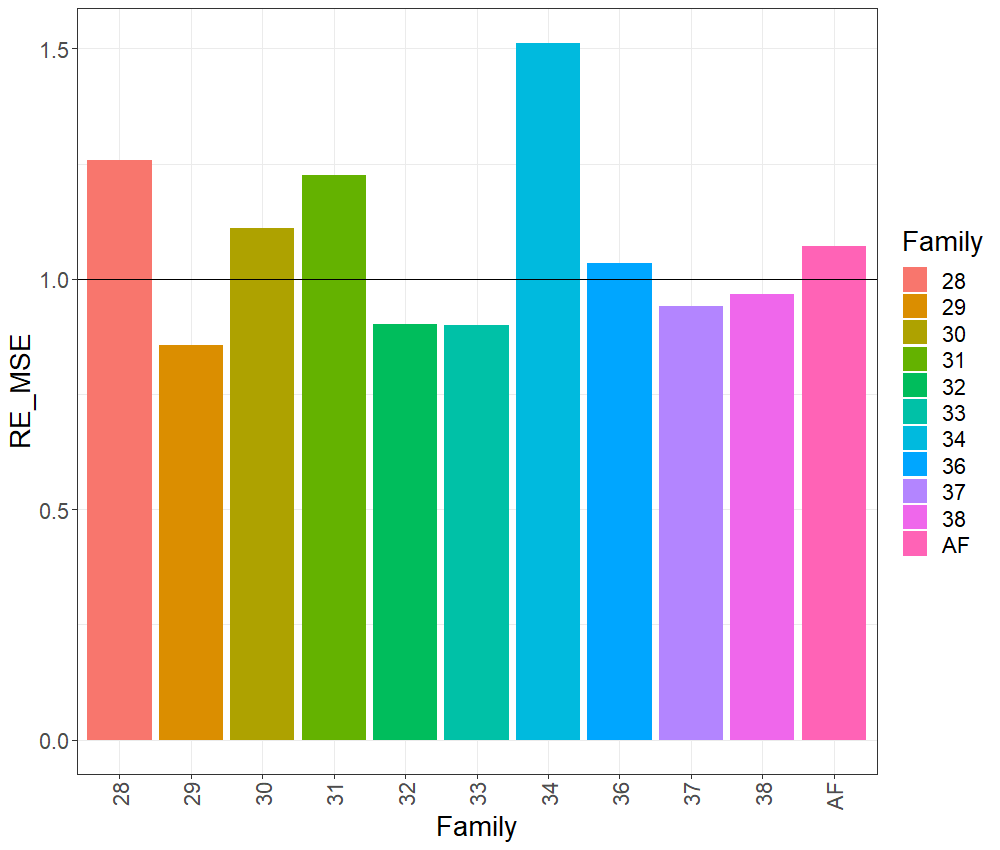 |
| **B** |
| 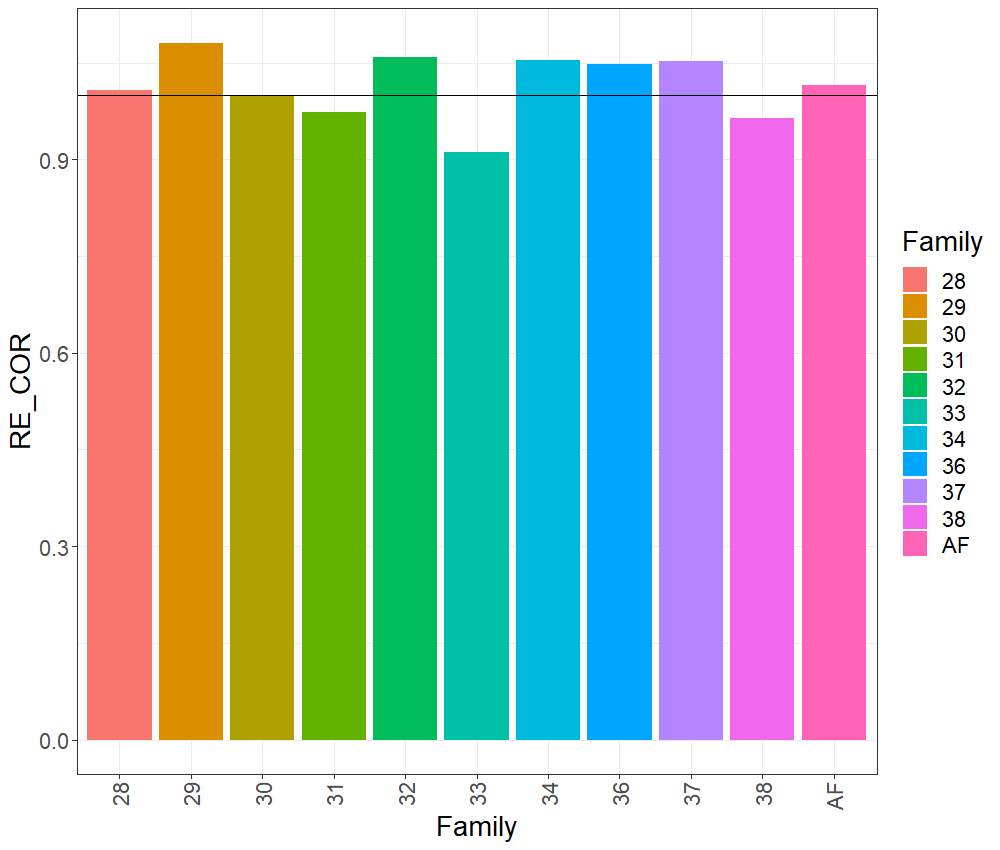 |
| **C** |
| 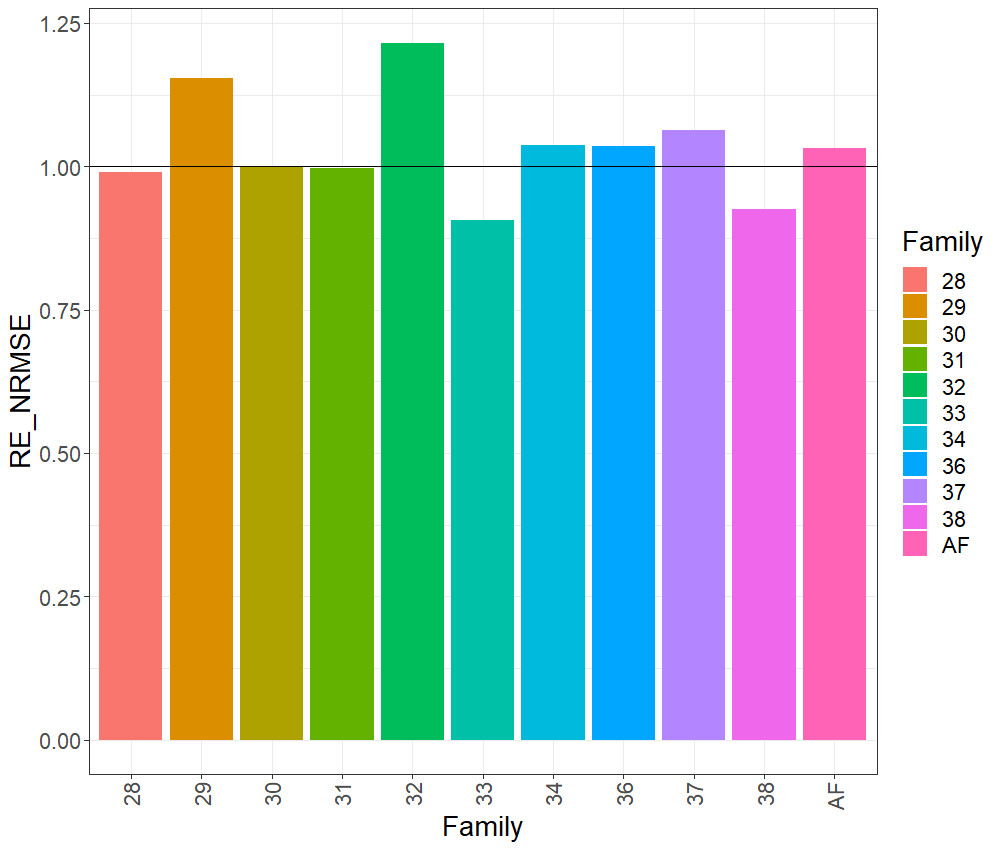 |

**Figure C5**. Data 8 (Soybean_3). Relative efficiency (RE) between the proposed method (AB, Adversarial validation with Boruta for variable selection) and the conventional, C, method for each family and across family (AF) in terms of A) Mean square error (MSE), B) Average Pearson´s Correlation (COR) and C) Normalized root mean square error (NRMSE). RE>1 means that the AB method outperformed the GBLUP method, RE<1 means that the GBLUP method outperformed the AB method and RE=1 means that both methods performed equally.

| **A** |
| --- |
| 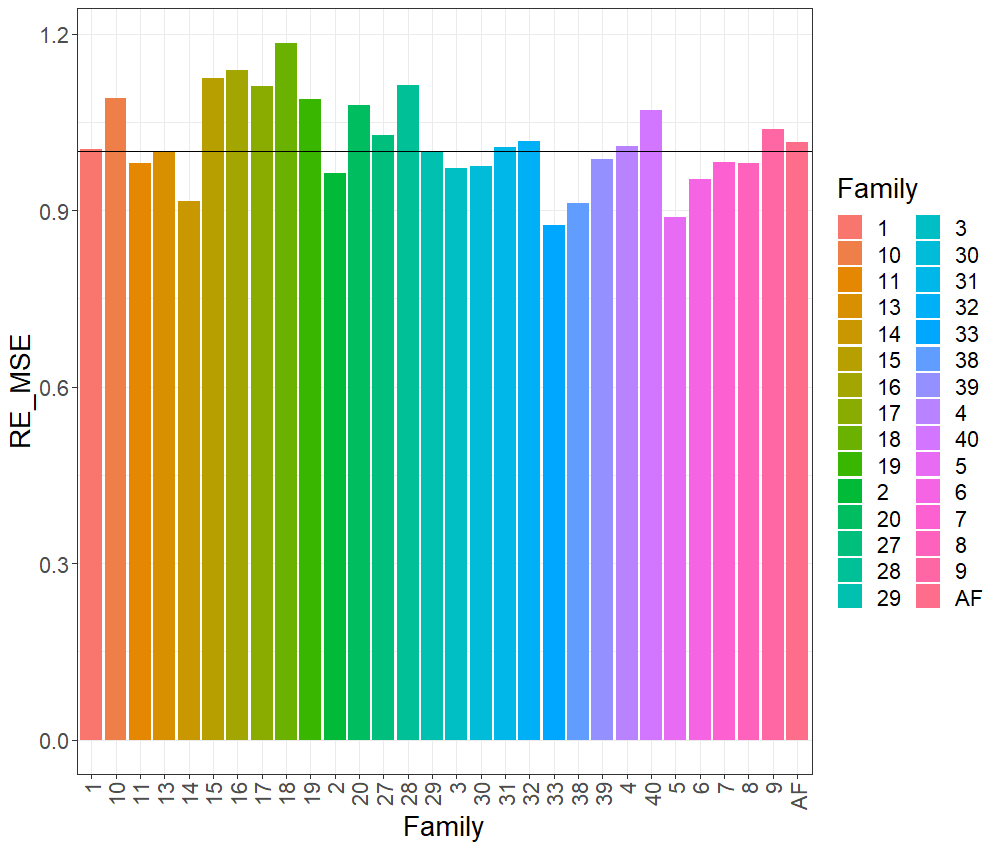 |
| **B** |
| 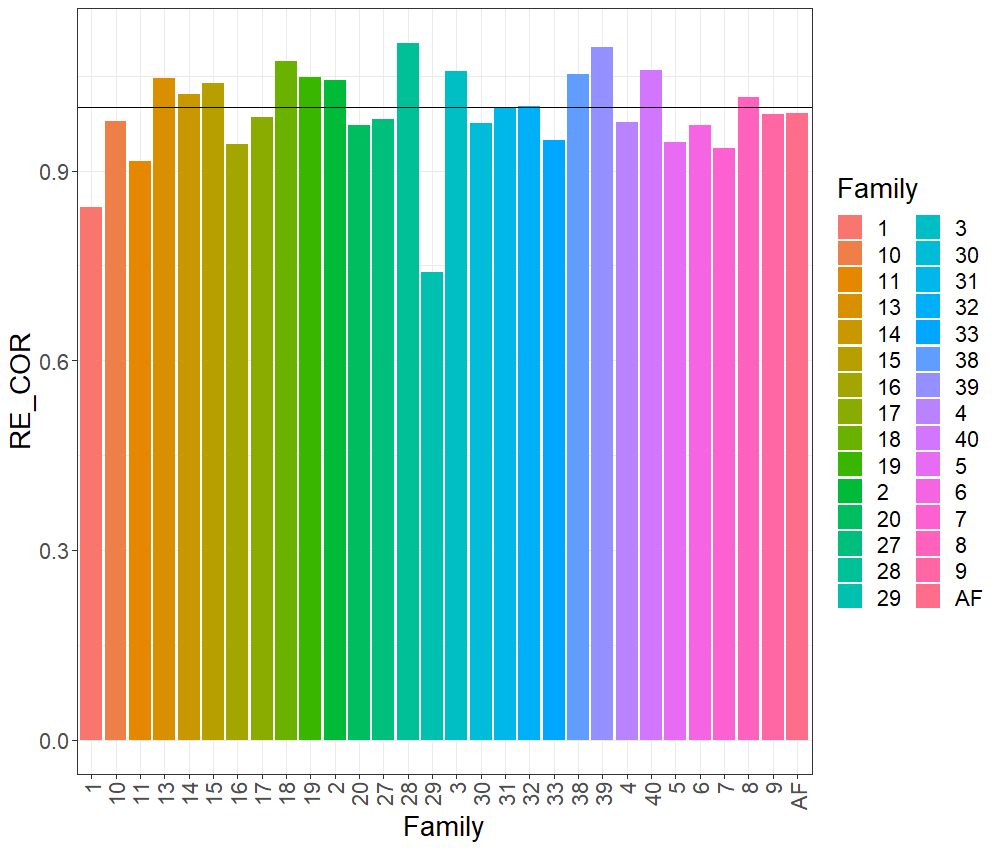 |
| **C** |
| 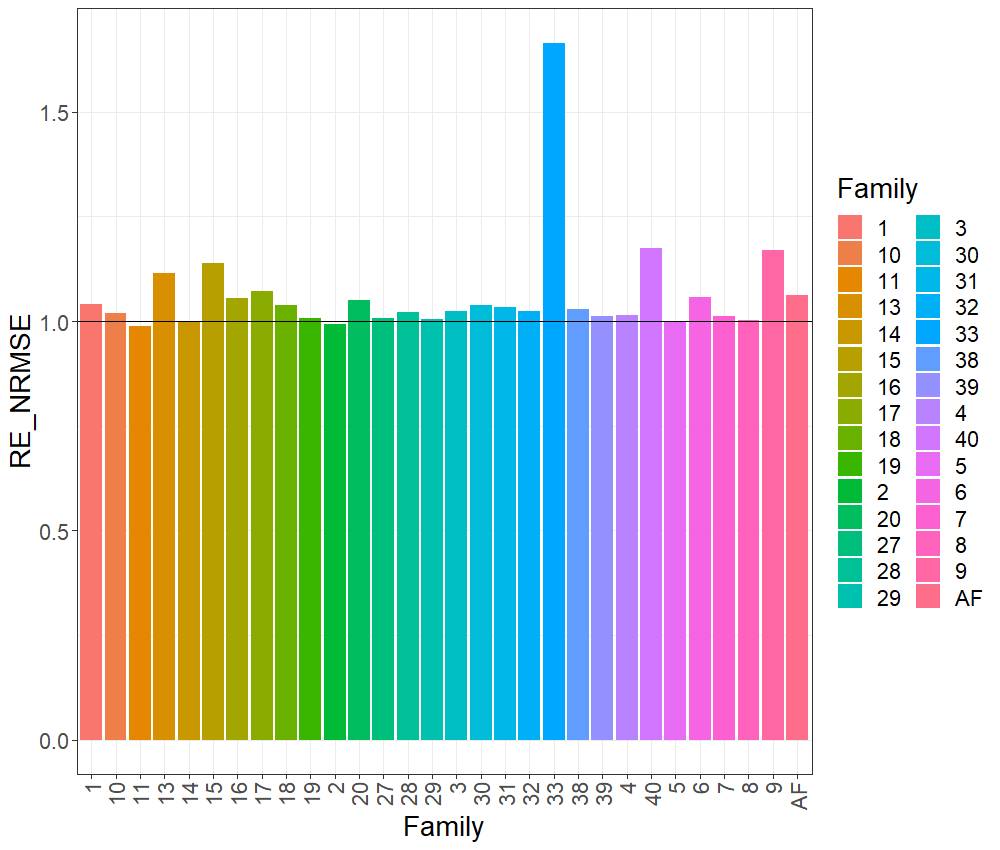 |

**Figure C6**. Data 10 (Maize_Bi_2018). Relative efficiency (RE) between the proposed method (AB, Adversarial validation with Boruta for variable selection) and the conventional, C, method for each family and across family (AF) in terms of A) Mean square error (MSE), B) Average Pearson´s Correlation (COR) and C) Normalized root mean square error (NRMSE). RE>1 means that the AB method outperformed the GBLUP method, RE<1 means that the GBLUP method outperformed the AB method and RE=1 means that both methods performed equally.

| **A** |
| --- |
| 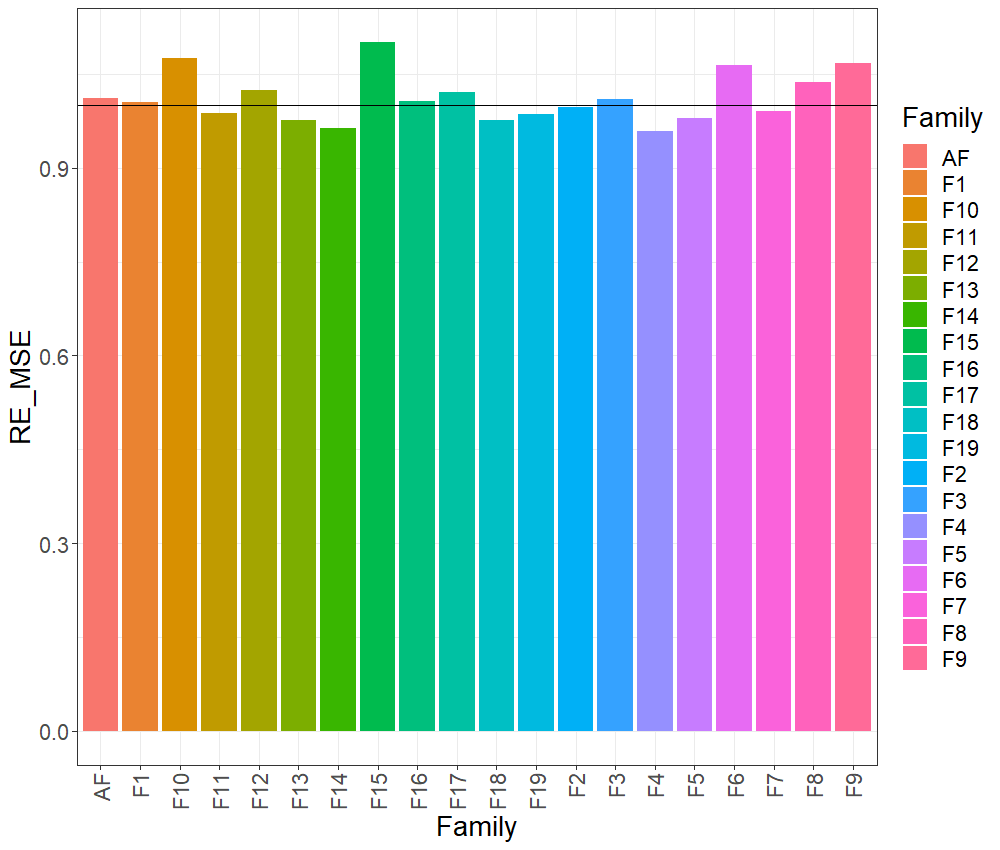 |
| **B** |
| 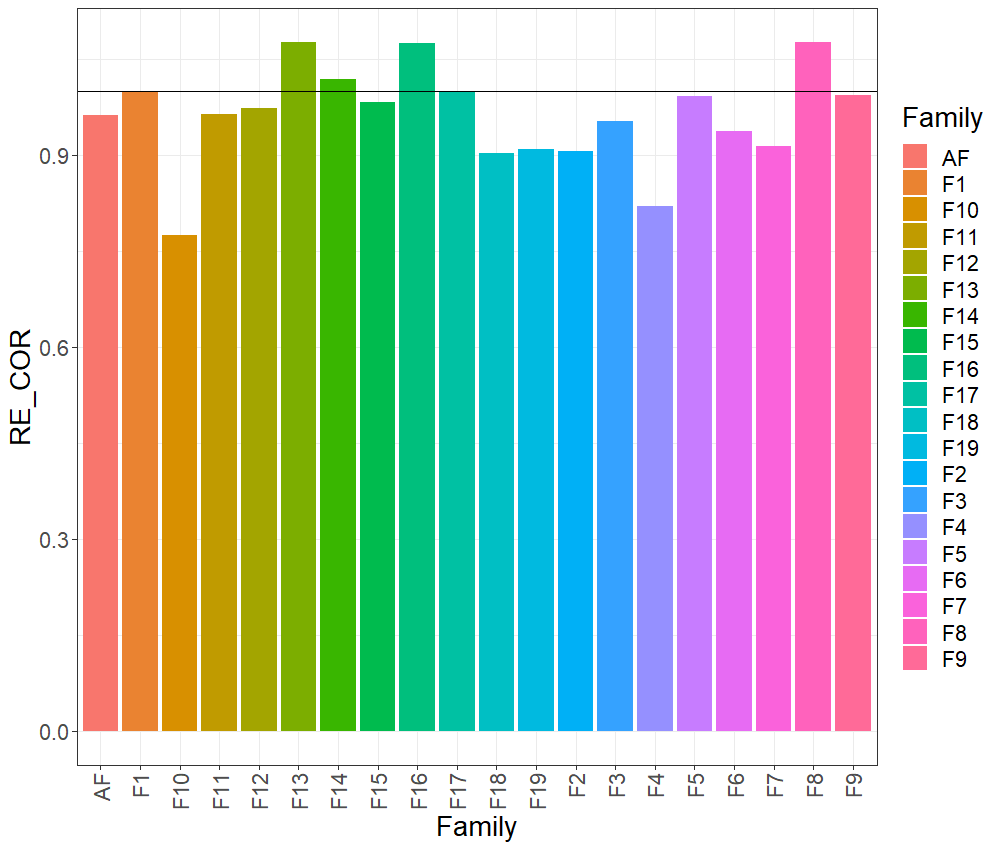 |
| **C** |
| 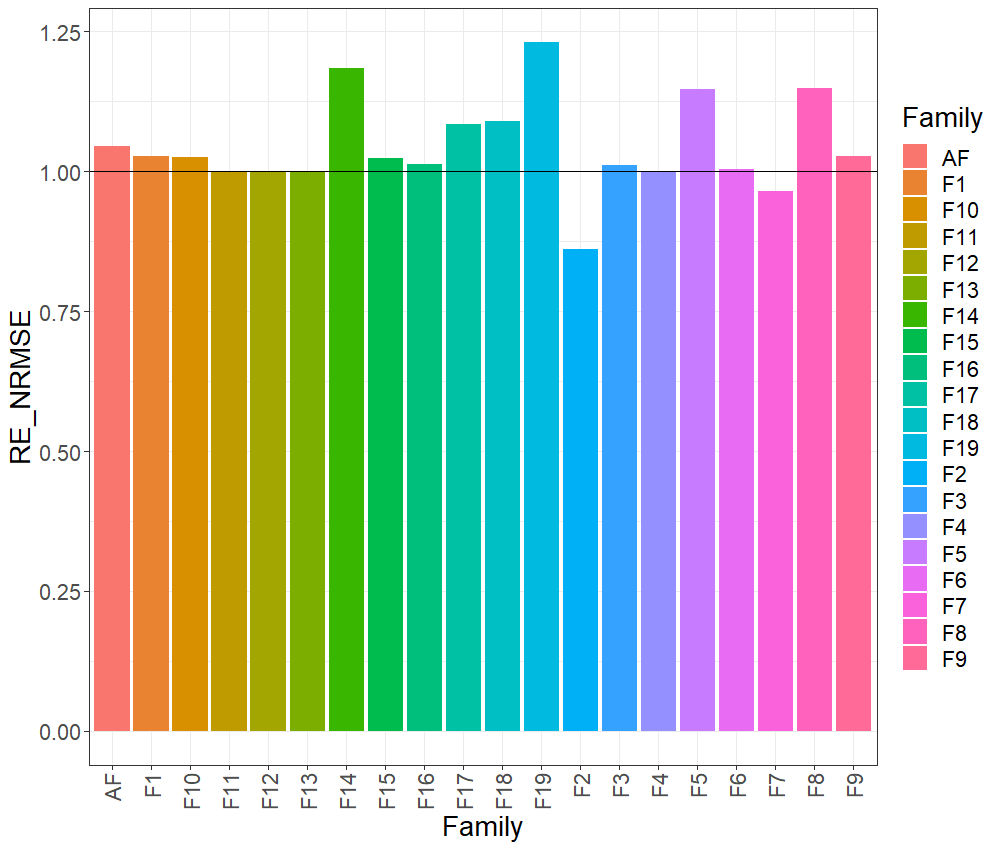 |

**Figure C7**. Data 11 (Maize_Bi_2019_D). Relative efficiency (RE) between the proposed method (AB, Adversarial validation with Boruta for variable selection) and the conventional, C, method for each family and across family (AF) in terms of A) Mean square error (MSE), B) Average Pearson´s Correlation (COR) and C) Normalized root mean square error (NRMSE). RE>1 means that the AB method outperformed the GBLUP method, RE<1 means that the GBLUP method outperformed the AB method and RE=1 means that both methods performed equally.

| **A** |
| --- |
| 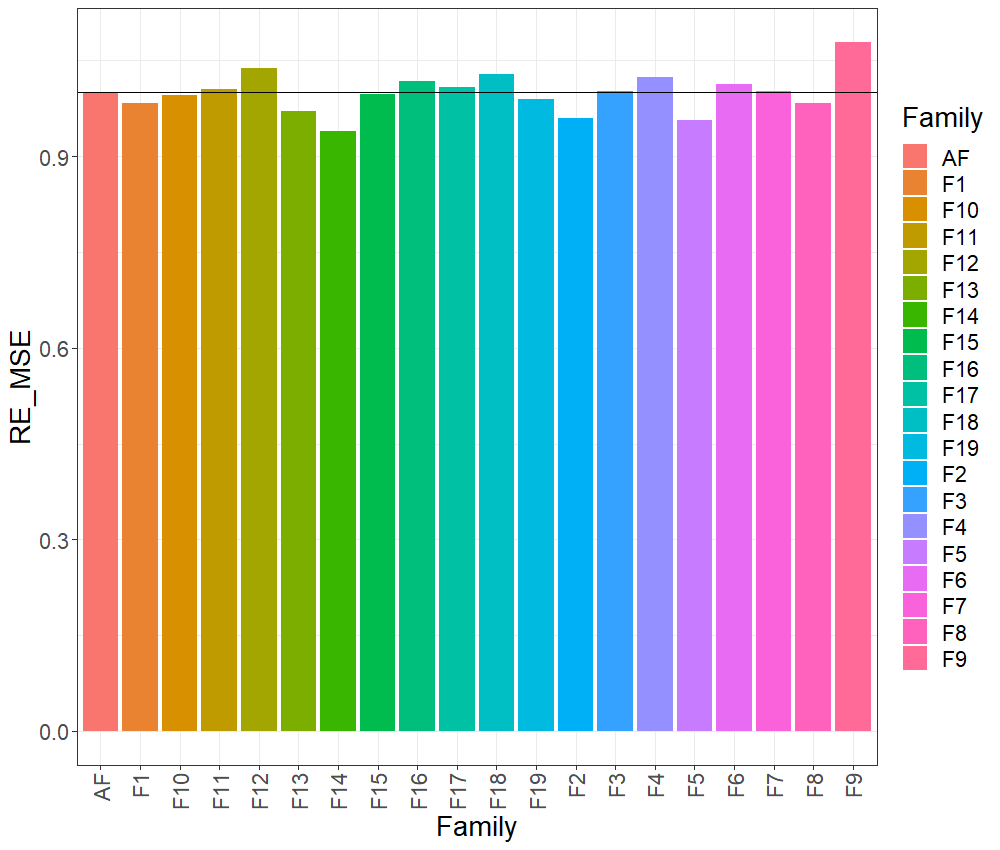 |
| **B** |
| 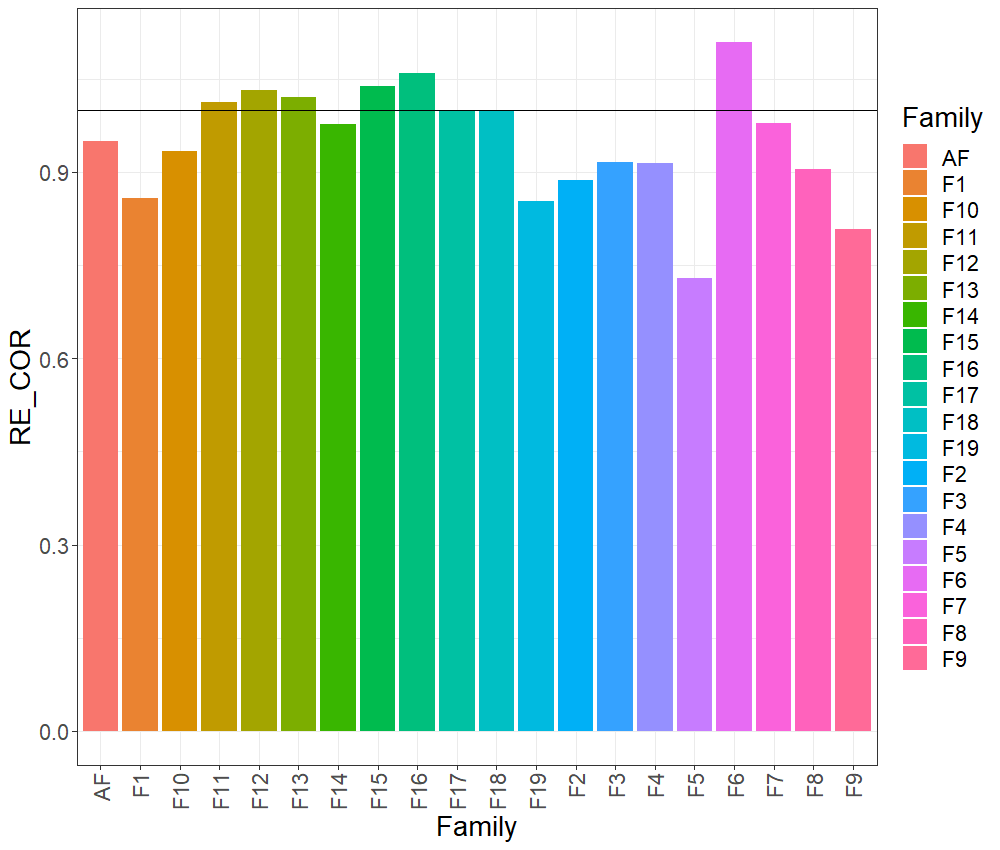 |
| **C** |
| 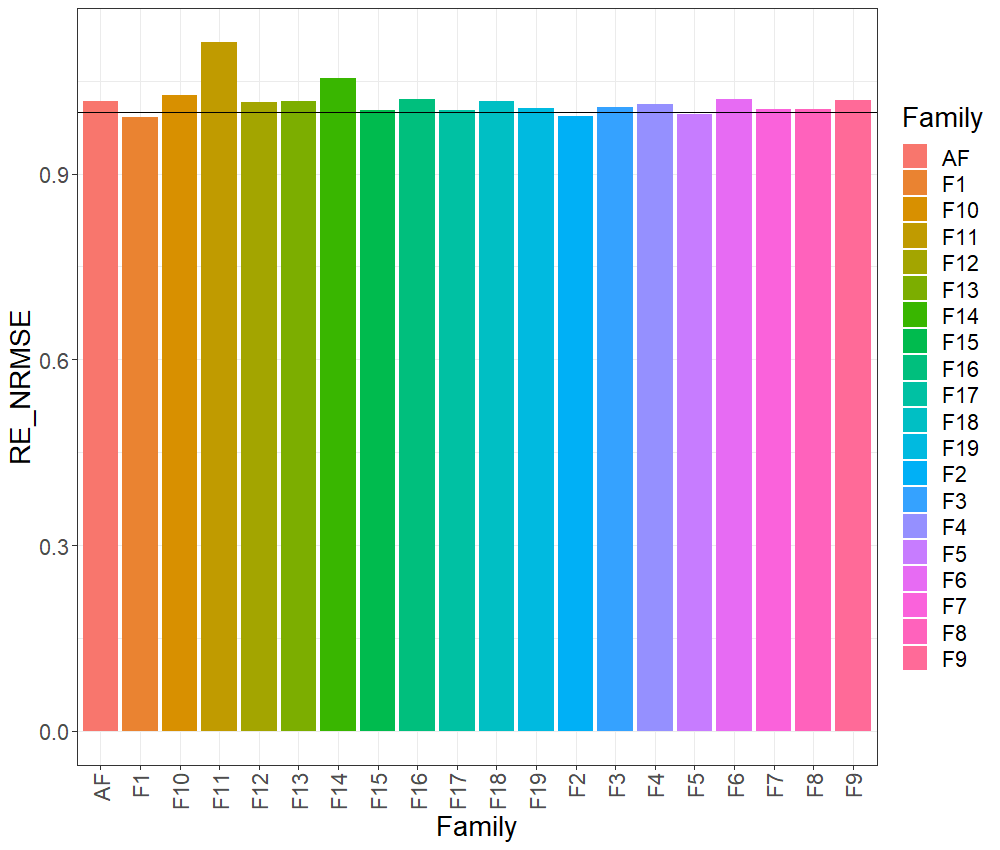 |

**Figure C8**. Data 12 (Maize_Bi_2019_O)**.** Relative efficiency (RE) between the proposed method (AB, Adversarial validation with Boruta for variable selection) and the conventional, C, method for each family and across family (AF) in terms of A) Mean square error (MSE), B) Average Pearson´s Correlation (COR) and C) Normalized root mean square error (NRMSE). RE>1 means that the AB method outperformed the GBLUP method, RE<1 means that the GBLUP method outperformed the AB method and RE=1 means that both methods performed equally.
